# A systematic review to assess the evidence-based effectiveness, content, and success factors of behavior change interventions for enhancing pro-environmental behavior in individuals

**DOI:** 10.3389/fpsyg.2022.901927

**Published:** 2022-09-05

**Authors:** Henriette Rau, Susanne Nicolai, Susanne Stoll-Kleemann

**Affiliations:** Chair of Sustainability Science and Applied Geography, University of Greifswald, Greifswald, Germany

**Keywords:** behavior change, intervention, pro-environmental behavior, climate change, sustainability, environmental psychology

## Abstract

To reduce global greenhouse gas emissions in order to limit global warming to 1.5°C, individuals and households play a key role. Behavior change interventions to promote pro-environmental behavior in individuals are needed to reduce emissions globally. This systematic literature review aims to assess the a) evidence-based effectiveness of such interventions and b) the content of very successful interventions without limiting the results to specific emitting sectors or countries. Based on the “PICOS” mnemonic and PRISMA statement, a search strategy was developed, and eligibility criteria were defined. Three databases (Embase, PsycInfo, and Web of Science) were searched to retrieve and review potential literature. As a result, 54 publications from 2010 to 2021 were included in the analysis. The results show that most interventions only have small positive effects or none at all. A total of 15 very successful interventions focused on the sectors of mobility, energy, and waste and incorporated improved (infra-) structures, education, feedback, enablement or made the sustainable option the default. Six evidence-based recommendations for content, timing, and setting are deducted and given for interventions on enhancing pro-environmental behavior (PEB). In summary, although the various interventions and intervention types to promote PEB differ in their effectiveness, very successful interventions have common elements. Future research should focus on high-/low-impact and high-/low-cost behavior to develop interventions that aim at high-impact but low-cost behavior changes, or avoid low-impact but high-cost behavior.

## Introduction

To reduce global greenhouse gas (GHG) emissions in order to limit global warming to 1.5°C, countries are now committed through agreements [e.g., 2030 Agenda for Sustainable Development (United Nations, 2015)] to work toward implementing sustainable living and consumption. For this purpose, individuals and households play a key role in climate-friendly transformations: “All households will need to play a part in this transformation” (HM Government, 2009, p. 82). The key focus is narrowed to pro-environmental behavior (PEB), which means “behavior that consciously seeks to minimize the negative impact of one's actions on the natural and built world” (Kollmuss and Agyeman, 2002, p. 240). To proactively take effective actions to minimize negative environmental impacts, a growing number of climate change experimentation and intervention studies to enhance PEB are conducted (Castán Broto and Bulkeley, 2013).

Peer-reviewed papers that describe tested interventions are included. The value added by this work is that the interventions and their contents are described and compared to each other in a comprehensible and novel way.

### Behavior change models

Behavior change is an important research topic in psychology, health, and sustainability sciences. Consequently, various models to effectively change behavior have been developed and discussed (Fishbein and Ajzen, 1975; Schwartz and Howard, 1981; Prochaska and DiClemente, 1984; Heckhausen and Gollwitzer, 1987; Bamberg, 2013; Steg et al., 2014). The most commonly used models to describe and encourage PEB (Hellbrück and Kals, 2012; Bamberg, 2013) are the theory of planned behavior [TPB, (Ajzen, 1991)] and the norm activation model [NAM, (Schwartz, 1977)]. However, both have been criticized for being oversimplified, too cognitive, and having a high empirical intention-behavior gap (Lewin et al., 1944; Hellbrück and Kals, 2012; Bamberg, 2013).

To address this criticism, the transtheoretical model (TTM, [Prochaska and DiClemente, 1984)] was used (mostly in the health domain but also in the sustainability context) to describe, explain, predict, and modulate intentional behavior change. The model consists of behavior change stages that need to be completed but also allows for several relapses to earlier stages before permanent behavior change is achieved. According to the TTM, interventions first need to focus on informing about and enhancing the positive aspects of behavior change to support the first stages and subsequently empower and reward behavior change during the last stages (Bamberg, 2007; Andersson et al., 2018).

However, TTM only considers intrinsic factors, which is why Gifford and Nilsson (2014) suggested a more complex expansion. The model of pro-environmental behavior that Kollmuss and Agyeman (2002) designed, specifically aimed at PEB, is an extended and complex model including external factors and feedback slopes. Their model was influenced by Fliegenschnee and Schelakovsky (1998), whose work was based on Fietkau and Kessel (1981). Both external factors (infrastructure, political and economic situation, etc.) and internal factors (personality traits, value system, etc.) influence PEB and are in return influenced by the individual's PEB—directly in the case of internal factors, and indirectly concerning external factors (Kollmuss and Agyeman, 2002). This complex model allows for various possible barriers to directly influence an individual's PEB. With the inclusion of external possibilities, the model clearly shows that individual behavior is only possible within a social framework, thereby limiting behavioral change. Interestingly, environmental consciousness, which belongs to the internal factors, consists of knowledge, emotional involvement, and values/attitudes, which can also serve as barriers and lead to negative feedback slopes (Kollmuss and Agyeman, 2002). This model was discussed and used as a theoretical basis in case studies, e.g., Stoll-Kleemann (2019) adapted this model to the practical issue of ocean-related PEB and thereby proved its applicability (Jensen, 2002; Payne, 2002).

In summary, even this modern, more specific, and extended model leads interventions to aim at increasing the knowledge base and both actively and emotionally involve individuals. Therefore, the models suggest that interventions should target several factors at once and not just gradually one after the other.

### Pro-environmental behavior change interventions

Abrahamse already listed well-used behavior change intervention types (Abrahamse, 2019), which allow researchers to categorize typical interventions as (1) providing (a) general or (b) tailored information/education; (2) providing feedback; (3) goal-setting/implementation intention and commitment; (4) nudging; (5) social influence, e.g., block leader, social modeling, social norms, or social marketing using message framing; (6) gamification; (7) policies, e.g., pricing or regulatory changes; and (8) structural measures, e.g., changing infrastructures.

According to Abrahamse (2019), the general approach to changing behavior is to provide information to enhance the knowledge base for decision-making. Information can either be general or tailored to specific situations or persons.

Giving individuals insights into their performance and the outcome of a certain task is called “feedback.” Consequently, feedback is used to increase a person's understanding of the relationship between performing a certain task (e.g., turning off appliances when not in use) and achieving a certain outcome (e.g., saving energy) (Cornelius et al., 2014; Abrahamse, 2019).

Additionally, setting a goal to achieve something (e.g., reduction of meat consumption in kg/week) or performing a task in a certain time frame can encourage behavior change. To support such goals, pledges can be made. These commitments can either be made verbally or in writing, and in private or public (Cornelius et al., 2014; Abrahamse, 2019).

Another intervention category is “nudging,” which as the word implies is a soft nudge toward behavior that is more desirable but not fully integrated into everyday life. Nudging interventions use small changes to the environment to influence the individual's decisions (Kurz, 2018; Abrahamse, 2019).

Furthermore, other people can also influence individuals and their behavior. For example, by observing the behavior of other people one can deduct what behaviors are expected from an individual in a certain situation and use it as a benchmark for one's own behavior. Another example is that social networks can be used to disseminate information, or that already familiar individuals can serve as role models, e.g., “block leaders.” Additionally, techniques like social marketing use and implement messages in specific ways to influence how people understand and respond to an issue, e.g., by triggering certain emotions or linking behavior to certain values that a person has (Schultz et al., 2007; Dolan et al., 2012; Abrahamse, 2019).

Interventions using typical game elements—like competitions, challenges, quests, and the possibility to earn badges/points and be compared to others *via* a leaderboard—outside of gaming contexts belong to the category of “gamification” (Cellina et al., 2019).

Another way to influence behavior is through policies. Policies are rules that people who belong to the area/group to which this policy applies have to adhere to. These policies can be local, national, or global.

Lastly, structural measures, which are mostly technological interventions, can be used to change the environment and encourage PEB. This could, for example, entail turning a street into a cycle and walking area while prohibiting cars, or installing water- and energy-saving appliances in a building.

In addition to the categorization made by Abrahamse (2019), Gardner and Stern (1996) differentiated between four major types of interventions: religious and moral approaches, education to change attitudes and provide information (similar to Abrahamse's “providing information”), changing the material incentive structure of behavior (similar to Abrahamse's “nudging”) and other types of rewards or penalties (similar to “structural measures”). They concluded that all of these intervention types can change behavior if they are executed carefully. In general, however, moral, and educational approaches, as well as incentive- and community-based approaches, were less effective. The most successful intervention types in the study by Gardner and Stern (1996) were combinations of various intervention types.

Recommendations made by Gardner and Stern (1996) as well as Stern (2000) include firstly identifying the target behaviors that have a significant impact on the environment. As a next step, the responsible actors and actions of that behavior should be identified. Afterward, the full range of causal variables needs to be considered. Also, the possible relevance to the target behavior from the actor's standpoint should be understood. Lastly, including the participation of representatives of the population whose behavior is to be changed enables interventions to become promising behavior change strategies (Gardner and Stern, 1996; Stern, 2000).

The most common methods to measure interventions' effectiveness include (a) reduced GHG emissions, (b) economic benefits, or (c) other societal gains. However, the (d) impact of interventions also needs to be considered by categorizing the “impact” as high or low based on the share of the emitting sector. The main sectors directly emitting anthropogenic GHG by economic segments are energy, i.e., electricity and heat production (25%), industry (21%), and transport/mobility (14%) as of 2010 (IPCC, 2014, p. 47). For individuals, the main GHG emitting sectors differ slightly from the economic range: mobility (34%), food (30%), housing (including electricity and heating) (21%), and others incl. consumption (15%) (Dubois et al., 2019). Additionally, the success of interventions can be measured as short vs. long-term behavior change and commitment.

However, Wynes et al. concluded that “it is unlikely that researchers have reached consensus on the most effective interventions” (Wynes et al., 2018, p. 5). Especially theoretical frameworks reach their limits when individuals deviate from rationality in their decision-making and resulting behavior (Frederiks et al., 2015). Consequently, empirical evidence is needed to identify effective behavior change interventions. While reviews regarding behavior change interventions exist (Abrahamse et al., 2005; Fisher and Irvine, 2016; Staddon et al., 2016; Andersson et al., 2018; Iweka et al., 2019; Stankuniene et al., 2020), these mostly (a) have used unsystematic or incompletely described methods, and/or (b) were limited to only one emitting sector or targeted level, i.e., (a) individual, (b) community, or (c) policy (intervention applies to whole business chain, or country-/union-wide). According to the targeted level, interventions can aim at changing (a) an individual's PEB, (b) a community's PEB, e.g., by changing infrastructure or social factors, or (c) policy-based PEB, e.g., prohibiting behavior with negative environmental impacts like overexploitation, or constituting/subsidizing PEB by replacing harmful technology.

A careful review of the literature indicates that the description of successful interventions' contents and/or combinations of interventions regardless of the emitting sector or targeted level do not appear to have been part of any reviews to date. For example, Stankuniene et al. (2020) conducted a review on behavioral barriers in households focusing on energy. Although the barriers are exhaustively defined, the content of the reviewed studies was not described in detail. Nisa et al. (2019) reviewed randomized controlled trials (RCT) on behavior change and found no evidence of sustained positive effects regarding PEB once the intervention ended. However, the reviewed studies seemingly only used single intervention types and, therefore, combinations of intervention types are not part of the review. Osbaldiston and Schott (Osbaldiston and Schott, 2012) did investigate combinations of intervention types and their effectiveness. However, they conclude “[…] that there is no one treatment (a ‘silver bullet') that is highly effective across all the possible PEB” (Osbaldiston and Schott, 2012, p. 280). Nisa et al. mentioned that “The content of interventions needs to be tested more precisely because a large proportion of interventions to date implements bundles of stimuli from which the identification of the key driver of effectiveness is difficult to grasp” (Nisa et al., 2019, p. 9).

This paper aims to close the research gap by systematically reviewing and analyzing the possible level of effectiveness of PEB interventions and the content of successful interventions based on evidence from the literature without limitation to only one specific GHG emitting sector or specific geographical units, e.g., countries. Using this approach, important implications for developing effective and appropriate interventions can be derived. Similar to the “what works” agenda in UK science-policy circles, this paper focuses on identifying and ranging effective, i.e., “successful,” intervention methods to provide recommendations concerning intervention development (West et al., 2019).

To assess the evidence-based effectiveness of behavior change interventions for enhancing PEB in individuals, this paper focuses on the following research questions:

1. Do effective interventions exist that increase PEB?
2. Do intervention types differ in their effectiveness to promote PEB?
3. Can recommendations be derived from evidence on how interventions for enhancing PEB should be designed and implemented?

## Materials and methods

This paper used a broad scope review, i.e., it is designed as a comprehensive summary of evidence to explore the consistency of the findings to date and to compare the impact or effectiveness of different interventions. Thus, it allows for generalizability across interventions.

### Systematic search methodology

Review methods developed within medical research, e.g., the Cochrane Handbook for Systematic Reviews of Interventions (Higgins and Green, 2019) as well as the PRISMA statement (Moher et al., 2009) can readily be applied to the sustainable research field and were used for this review. The aim is to enable others to reproduce the results, and thereby make the review more usable for decision-makers.

For this purpose, the “PICOS” mnemonic was used to formulate the database query. PICOS is an acronym for the elements Problem/Population, Intervention, Comparison(s), Outcome(s), and Setting (Moher et al., 2009). The review's PICOS set the inclusion and exclusion criteria of PEB-intervention studies.

To capture the quite dispersed range of literature in this field, the queries used in the search strategy were chosen to cover the three main GHG emitting sectors (mobility, energy/housing, food) responsible for 56–85% of individuals' GHG emissions (Bundesministerium für Umwelt, 2018; Dubois et al., 2019). This made the search as specific as necessary while being as generic as possible. However, to not limit this review to predefined emission sectors but also include uncommon interventions or interventions aiming at other emission sectors, publications with data of PEB-interventions regarding other sectors, e.g., consumerism, were also considered eligible and included in this review if such were identified by the search queries.

The already defined search terms (see Table 1) within a PICOS element were linked *via* the Boolean operator “OR.” This means that a resulting publication should at least contain one search term per PICOS element. The elements A to E were connected with AND operators.

**Table 1:** Search terms for the systematic literature review using the “PICOS” mnemonic.

**PICOS element**	**Search terms**
(A) Problem	Carbon footprint; CO^*^Fu^*^abdruck; green house gas; GHG; Treibhausgas; emission
(B) Intervention	Experiment; intervention; behavio^*^ change; Verhaltensänderung; Verhaltensveränderung; household decision-making; transition; value system^*^; Wert^*^system; habit; Gewohnheit; dissociation; dissonance; Dissoziation; Dissonanz
(C) Comparisons	Energy; energy consumption; Energie; Energieverbrauch; mobilit^*^; transport; air travel; travel decision; Ernährung; diet
(D) Outcomes	Motivation; self-efficacy; self-identity; ^*^environmental ^*^identity; Identität; pro-environmental behavio^*^; environmental awareness; sustainab^*^; mitigation; adaptation; sufficiency; Nachhaltigkeit^*^; Anpassung; Suffizienz; low^*^cost; high^*^cost; incentive; income; Anreiz; Einkommen
(E) Setting	Climate change; climate crisis; Klimawandel; Klimakrise; environmental change; planetary boundaries

* Is used as a wildcard and stands for any character(s) that might occur enabling the database query to search for words with different possible spellings, e.g., “behavior” in British or “behavior” in American English.

Comprehensive literature searches to identify eligible intervention studies were conducted on 1 April 2020 using multiple academic online databases and their database-specific syntaxes namely, Embase, Web of Science, APA PsycInfo, APA PsycArticles, and Psychology as well as the Behavioral Sciences Collection *via* EBSCOhost (see Supplement “Database Searches,” Supplementary Tables 1–3). This comprehensive literature search was continuously updated until 1 March 2022 using search alerts for the respective databases. Only relevant and accessible publications were retrieved from the search alerts and included in the category “identified through other sources” to not compromise the reproducibility and results of the original systematic literature search.

### Eligibility criteria

The eligibility criteria are based on the PICOS elements of the review question plus a specification of the types of studies that have addressed these questions.

Publications were considered eligible when published in English or German, from 2010 on, and available in full text. The included publications should be peer-reviewed and needed to be a study of a behavior change intervention in relation to PEB, which reported on outcomes generated through the intervention. The intervention design should allow for effects to be measured either compared to a baseline (pretest/post-test design) or a control group. Eligible studies could report on interventions in any country and for any emission sector or targeted behavior, as long as the methodology and outcomes are described in a comprehensible manner without apparent quality problems. Existing reviews were included if they provided information on the study population, intervention methodology, and outcomes of at least one intervention study that was otherwise unavailable in full-text to the authors.

Publications were not considered eligible when they focus on (a) models, e.g., to compute savings or possible scenarios; (b) (discrete choice or framing) experiments analyzing the intention or willingness of participants only; (c) measuring or quantifying GHG emissions without intervention; (d) assessing technical solutions only. Whilst technologies and intention-based experiments create a new context in which behavior change can take place, these do not aim to induce changes in behavior. Consequently, such publications were not considered in this review. Excluded papers from full-text screening including the reason for exclusion can be found in the Supplementary material under “Excluded Publications”.

### Selection process

The titles and abstracts from the preliminary search were retrieved and reviewed for relevancy and the full-text articles of relevant studies were retrieved, if possible, for further review. The retrieved full-text articles were assessed for inclusion based on the criteria listed above and inconsistencies were resolved between the authors during all review phases. A table summarizing the included studies was prepared (see Table 2), with the following segments: country of conducted intervention, GHG emitting sector the intervention is targeting, type and description of intervention approach, the effectiveness of the intervention, and targeted level.

**Table 2:** Summary of included publications.

**Publication**	**Country**	**Sector**	**Intervention type**	**Effectiveness (++ / + / o / –)**	**Level**
Aiken (2018)	UK	Energy	Social influence *via* motivational interviewing, energy audits, and reimbursement	+	Community
Araña and León (2016)	Spain	Mobility and consumption (tourism)	Social influence *via* message framing, time pressure, or pricing policy—RCT using a field experiment with fully consequential choices	+	Policy
Asensio and Delmas (2015) (in Wynes)	See Wynes (USA)	See Wynes (energy)	See Wynes (information and feedback campaign integrating social marketing messages and non-price incentives—RCT incl. survey data)	See Wynes (+ overall, ++ in subgroup with children)	See Wynes (individual)
Barata et al. (2017)	Portugal	Energy and water	Information and feedback campaign—controlled trial incl. survey data	+	Individual
Beitzen-Heineke et al. (2017)	Germany, Austria, Italy	Consumption	Structural measures—zero packaging grocery stores as an alternative retail concept	+	Community
Benka-Coker et al. (2018)	Ethiopia	Energy	Structural measures—provision of technology, i.e., less GHG-emitting ethanol cookstoves in (a) a Refugee program and (b) low-income urban intervention	+	Community
Börner et al. (2015)	Netherlands	Energy	Information campaign	+	Individual
Bohdanowicz et al. (2011)	Europe	Energy, water, waste	Policies regarding the sustainability of a hotel chain	+	Policy
Boso et al. (2019)	Chile	Energy	Structural measures—governmental replacement program to replace 39,000 wood-burning stoves in the municipalities of Temuco and Padre Las Casas by 2020	+	Policy
Brand et al. (2014)	UK	Mobility	Structural measures—new walking and cycling infrastructure	o	Community
Büchs et al. (2018)	UK	Energy	Tailored information trial using a carbon calculator interview (RCT)	o	Individual
Carrico and Riemer (2011) (in Staddon)	See Staddon (USA)	See Staddon (energy)	See Staddon (General information, peer education, and feedback)	See Staddon (+)	See Staddon (community)
Casals et al. (2020)	UK	Energy	Gamification	+	Individual
Cellina et al. (2019)	Switzerland	Mobility	Gamification including feedback and social influence (comparison); RCT study design	o (+ in systematic routes in one region)	Individual
Chiu et al. (2020)	Taiwan	Energy	Feedback and social influence (persuasive technology, comparison with peers, and rewards)	+	Individual
Cornelius et al. (2014)	USA	Energy, food, mobility	Information, goal setting, social influence, gamification, feedback in a classroom setting; cluster-RCT study design	+	Community
Damsø et al. (2017)	Denmark	Energy	Policies and structural measures like renewable energy provision as a default for a municipality	++	Policy
Dawkins et al. (2019) (Review)	OECD-countries	Consumption	Policies and structural measures	o /+ (depending on the study)	Policy
Dowd et al. (2012)	Australia	Energy	Social influence incl. tailored information, goal setting, and feedback for low-income participants	++	Individual
Fisher and Irvine (2016) (Review)	Netherlands (*N* = 1), UK (*N* = 3)	Energy	Social influence incl. information, commitment, and feedback	++	Individual
Hall et al. (2013)	Australia	Energy	Social influence incl. general information	+	Individual
Hammed et al. (2018)	Nigeria	Waste (recycling)	Social influence incl. practical education	++	Community
Happer and Philo (2016)	UK	Not specified	Social influence *via* media messages	o	Individual
Hoicka et al. (2014)	Canada	Energy	Policies and structural measures—provision of audit/tailored information and technical solutions/retrofit improvements for house owners (incl. reimbursement in programs 2 and 4)	++	Individual
Howarth and Roberts (2018)	UK	Energy	Policies and structural measures—provision of audit/tailored information and suggestions for technical solutions/retrofit improvements to households incl. governmental incentive structure (paying back a loan attached to the house *via* energy bill)	o	Individual
Howell (2011)	UK	Energy, food, mobility	Social influence *via* message framing using a movie	o	Individual
Howell (2012) (in Fisher)	See Fisher (UK)	See Fisher (energy)	See Fisher (Social influence incl. information, commitment, and feedback)	See Fisher (++)	See Fisher (individual)
Iweka et al. (2019)(Review)	See Fisher, others not specified	Energy	General information (energy labels, prompts), tailored information (energy audits), social influences (norms, block leader), goal setting and commitments, feedback, gamification, incentives	o (general information, incentives) + (tailored information, norms) ++ (commitments, goal setting, feedback, gamification, block leaders)	Individual, community
Jacobsen et al. (2013)	USA	Energy	Policies and structural measures—state-sanctioned, green-electricity options program to fund the development of renewable energy systems incl. commitment (“municipality pledge”) and earning points for residential signups to earn incentives for the community (solar panels)	++	Individual, community
Jorgensen et al. (2021)	Australia	Energy	Information—two experiments with students in residential halls (as consumers, who are not billed for energy consumption) with information about reducing energy consumption during the peak demand period—additionally, experiment 1 used normative feedback, and experiment 2 used normative feedback and prompts/reminder notifications at different time points, RCT study design.	+	Individual, community
Keall et al. (2015)	New Zealand	Mobility	Structural measures—public investment in infrastructure for walking and cycling plus social influence (social marketing) compared with a control group (two other cities with similar characteristics)	+	Individual
Kelly et al. (2013) (Review)	Not specified	Food	Social influence, goal setting, feedback, general information; six studies were RCTs	o/+ (depending on the study, changes were not maintained at follow-up)	Individual
Kurz (2018)	Sweden	Food	Nudging (changing menu order and visibility of dish—vegetarian dish was moved to the top of the menu and visible at the point of decision-making) compared to control restaurant with similar characteristics	+ (positive change maintained even 13 weeks after intervention)	Individual
Laakso (2017)	Finland	Mobility	Social influence and commitment—household has to sell one car and receives free travel cards for local (bus) services incl. survey data and follow-up	++ (the positive change was mostly maintained at follow-up)	Individual
Largo-Wight and Wight (2013)	USA	Waste (recycling)	Structural measures and nudging—adding indoor opportunities to recycle cans and bottles compared with only-outdoor-receptacles control	++	Individual
Malan et al. (2020)	USA	Food	Education—one-unit seminar course “Foodprint seminar” at universities regarding food systems and sustainability incl. surveys to assess climate change self-efficacy amongst other things	+	Individual, community
Marchand et al. (2015)	UK	Energy	Policies and structural measures—provision of audit/tailored information and suggestions for technical improvements to households incl. free of charge installation	+	Individual
Matsui et al. (2014)	Japan	Energy	Feedback on electricity usage and general information compared to control	o (- for one household because of their changed circumstances during intervention)	Individual
Meloni et al. (2011)	Italy	Mobility	Policies with an activity-travel-measurement app and personal weekly maximum amount of personal carbon emissions (cap) incl. commitment and general information	+	Individual
Morris et al. (2016)	Australia	Energy	Policies and structural measures—solar city project; social influences (in-home energy assessment incl. tailored information and free-of-charge replacements/installations), commitment, gamification (competition), and residents asked to host solar panels of power providers on their roofs to reduce emissions without direct benefit	++	Individual, community
Mrkajic et al. (2015)	Serbia	Mobility	Structural measures—providing a secure bicycle parking facility incl. survey data, comparison with control	+	Individual
Nishida et al. (2016)	Japan	Energy	Policies—Cap-and-Trade Program for energy consumption-related emissions in buildings incl. goal setting and social/organizational approaches (performance disclosure and certification system)	++	Policy
Ornaghi et al. (2018)	UK	Energy	Social influence—motivating messages incl. general or tailored information about window management during the heating season compared to control with similar characteristics	+ (changes maintained at follow-up for tailored information)	Individual
Quested et al. (2013)	UK	Food	Social influence incl. education—Love Food Hate Waste messages, recipes, tips, and training to encourage the use of food rather than a reduction in waste	+	Individual, community
Reeves et al. (2014)	UK	Energy, food, mobility, consumption	Social influence—Support for community-led actions and initiatives	+	Community
Revell (2014)	UK	Energy	On-site energy audits incl. tailored information, installing easy measures and suggestions for further technical improvements for households plus survey data	+ (but no actual behavior change)	Individual
Ro et al. (2017) (in Iweka)	USA	See Iweka (energy)	See Iweka (Gamification incl. online game, receiving credits for completed tasks, team competition, leaderboard)	See Iweka (+) (+ and positive behavior changes maintained at follow-up)	See Iweka (individual)
Ruiz-Tagle and Schueftan (2021)	Chile	Air pollution	Nudging and information—information sign as a magnet above the stove's setting, including visits/phone calls to households; RCT incl. survey data)	+	Individual
Schultz et al. (2015)	USA	Energy	Social influence (social marketing)—in-store and school-based events to make people purchase and install LED light bulbs, incl. commitment, education, and rebates compared to control stores	+ for electricity consumption behavior change	Individual
Sintov et al. (2016)	See Wynes (USA)	See Wynes (energy)	See Wynes (Gamification [competition with incentives], general information, and feedback on electricity usage plus survey data)	See Wynes (+)	See Wynes (individual)
Staddon et al. (2016) (Review)	USA (*N* = 10), UK (*N* = 5), Netherlands (*N* = 3), Canada (*N* = 2), Sweden (*N* = 1), and Singapore (*N* = 1)	Energy (*N* = 22)	Social influence *via* volunteers in workplace, meetings, general or tailored information and education, feedback, goal setting, gamification (competition and challenges), structural measures (e.g., building renovation)	++ (eight studies), + (12 studies)	Individual, community
Wang and Boggio-Marzet (2018)	Spain	Mobility	Education—eco-driving training to apply emission and fuel consumption-reducing techniques	+	Individual
West et al. (2019)	UK (*N* = 1), Sweden (*N* = 1)	Energy, food, consumption, mobility	Tailored information using a Carbon Footprint Calculator	o	Individual
Wynes et al. (2018) (Review)	USA (*N* = 15), Denmark (*N* = 4), Sweden (*N* = 3), China (*N* = 1), India (*N* = 1), South Africa (*N* = 1)	Energy (N = 29), food (N = 6), mobility (N = 5)	Nudging (food, energy), information, feedback, social influences incl. goal setting, commitment, gamification (competition)	+/++ depending on the study	Individual

Effectiveness is reported in Table 2 as a positive value when the intervention group changed its behavior to increased PEB compared to baseline values or a control group (+)—or in case of significant increase (++)—and a negative value when the intervention led to decreased PEB (-). In case the intervention did not affect actual behavior the intervention is rated with an “o.”

### Reducing risk of bias

To reduce the risk of overestimating the effects of interventions, intervention studies are only considered once in the analysis. This means that all relevant publications of interventions are included in the quantitative results of the literature search. However, if they are also part of a review that is also included in the analysis, they will only be considered as part of the review. Consequently, these publications will be included in Table 2 with a respective remark and only reviewed as full-text to ensure the extraction of the most information possible.

## Results

**Figure 1:**
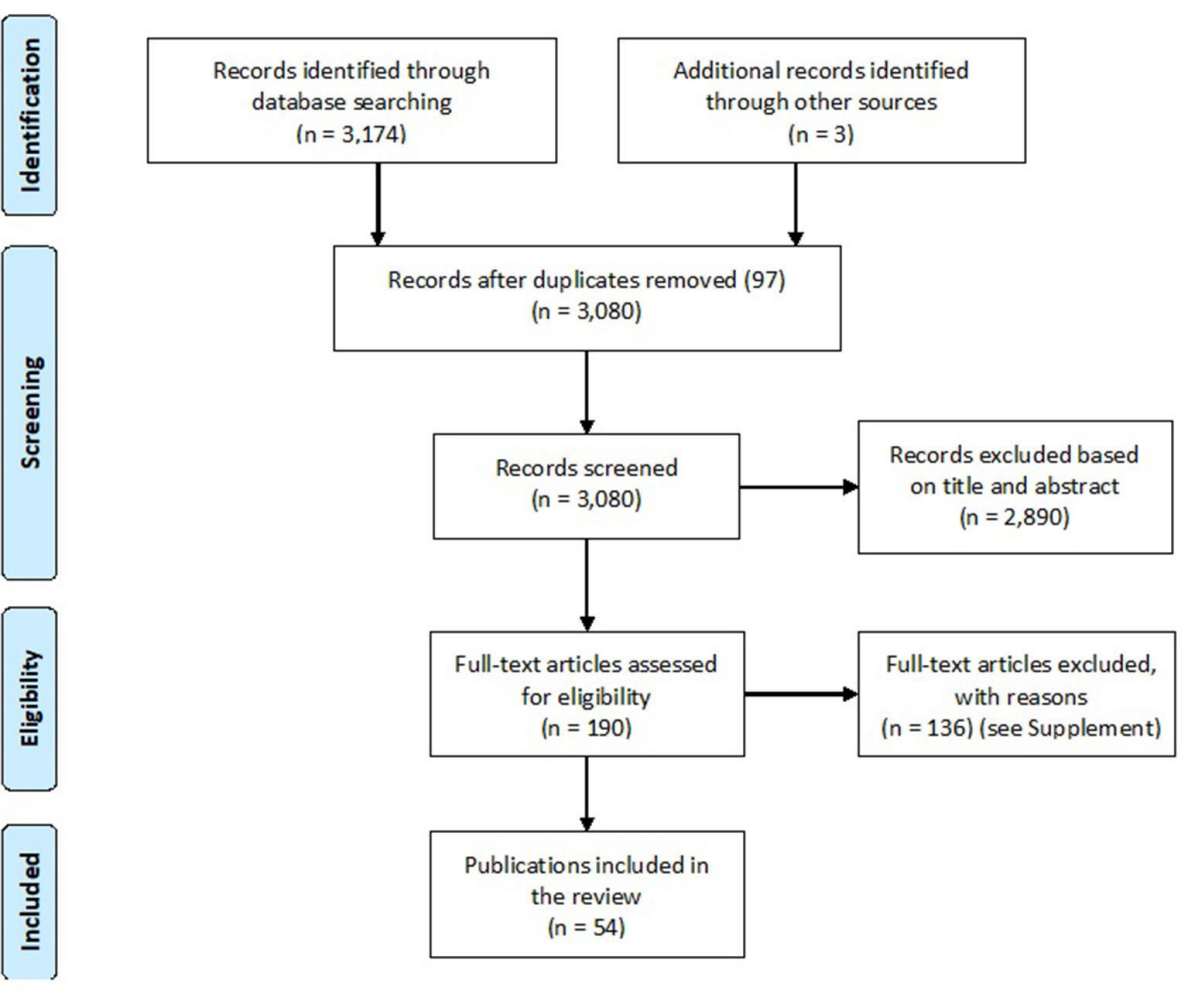
PRISMA flow chart of the conducted literature search [own representation based on Moher et al. (2009)].

**Figure 2:**
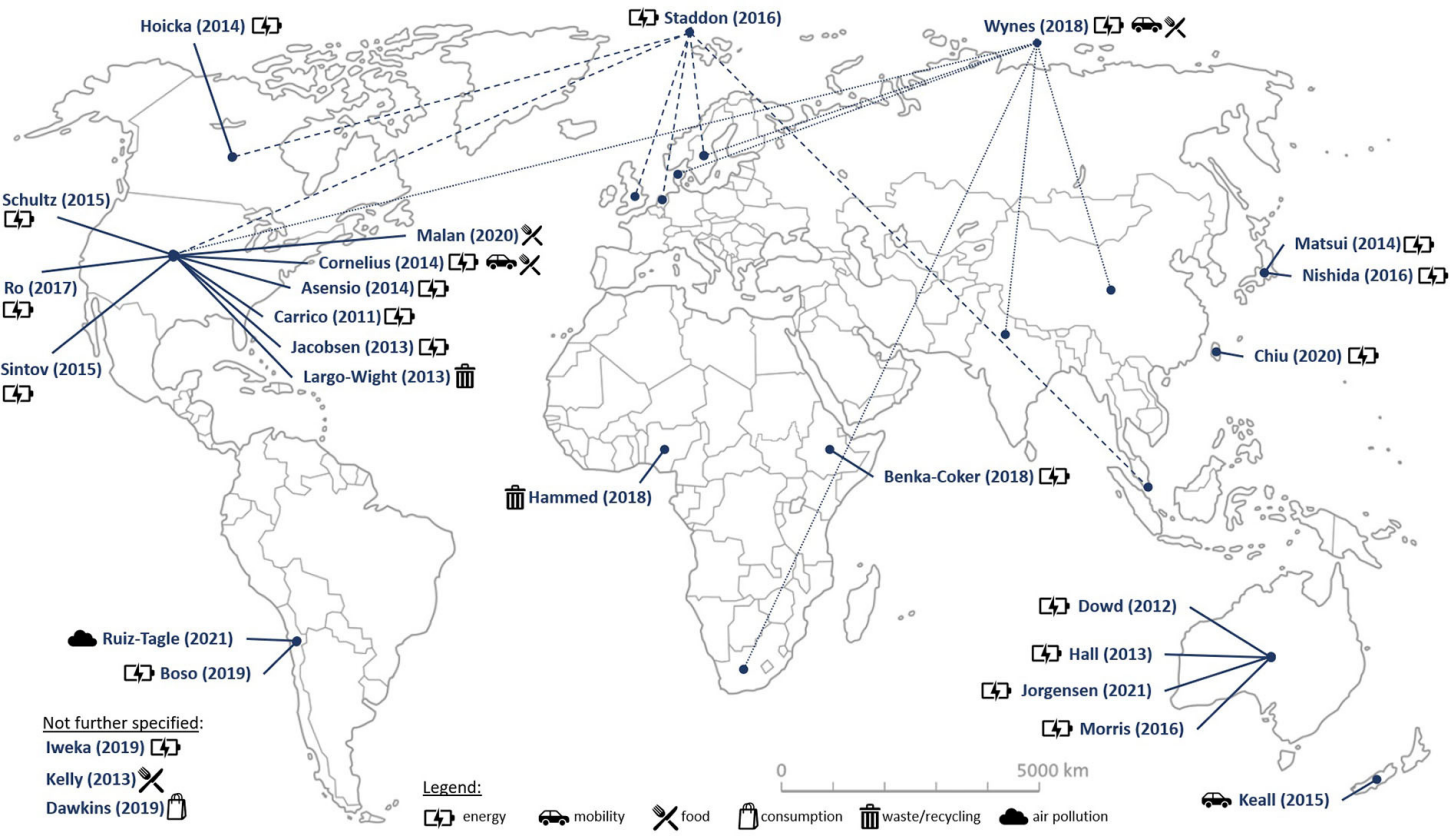
Worldmap as graphical summary of the results (except studies only conducted in Europe) regarding location and sector, which the intervention studies aimed at (own illustration).

The database searches resulted in a total of 3,174 publications using Embase (*n* = 162), APA PsycInfo, APA PsycArticles and Psychology as well as the Behavioral Sciences Collection *via* EBSCOhost (*n* = 79), and Web Of Science (*n* = 2,933) (see Supplementary material “Database Searches”). Additionally, search alerts were used from 2 April 2020 until 1 March 2022 to identify further publications. These search alerts suggested a further 1,657 publications (Embase *n* = 91, EBSCOhost *n* = 19, Web Of Science *n* = 1,547), including duplicates, resulting in three publications, which were included as “identified through other sources” in this review. After screening and assessing the full texts for eligibility, 54 publications were included in this review (see Figure 1). All publications were published in English, dating from 2010 to 2021. As depicted in Figure 2, four of the described studies in these publications were conducted in Australia, one in New Zealand, three in Africa (one each in Nigeria, Ethiopia, and South Africa), and six in Asia (one each in China, India, Singapore, and Taiwan, and two in Japan), 35 in North America (USA *n* = 32, Canada *n* = 3), and two in South America (Chile *n* = 2) (Carrico and Riemer, 2011; Dowd et al., 2012; Hall et al., 2013; Jacobsen et al., 2013; Largo-Wight and Wight, 2013; Cornelius et al., 2014; Hoicka et al., 2014; Matsui et al., 2014; Asensio and Delmas, 2015; Keall et al., 2015; Schultz et al., 2015; Morris et al., 2016; Nishida et al., 2016; Sintov et al., 2016; Staddon et al., 2016; Ro et al., 2017; Benka-Coker et al., 2018; Hammed et al., 2018; Wynes et al., 2018; Boso et al., 2019; Chiu et al., 2020; Malan et al., 2020; Jorgensen et al., 2021; Ruiz-Tagle and Schueftan, 2021).

**Figure 3:**
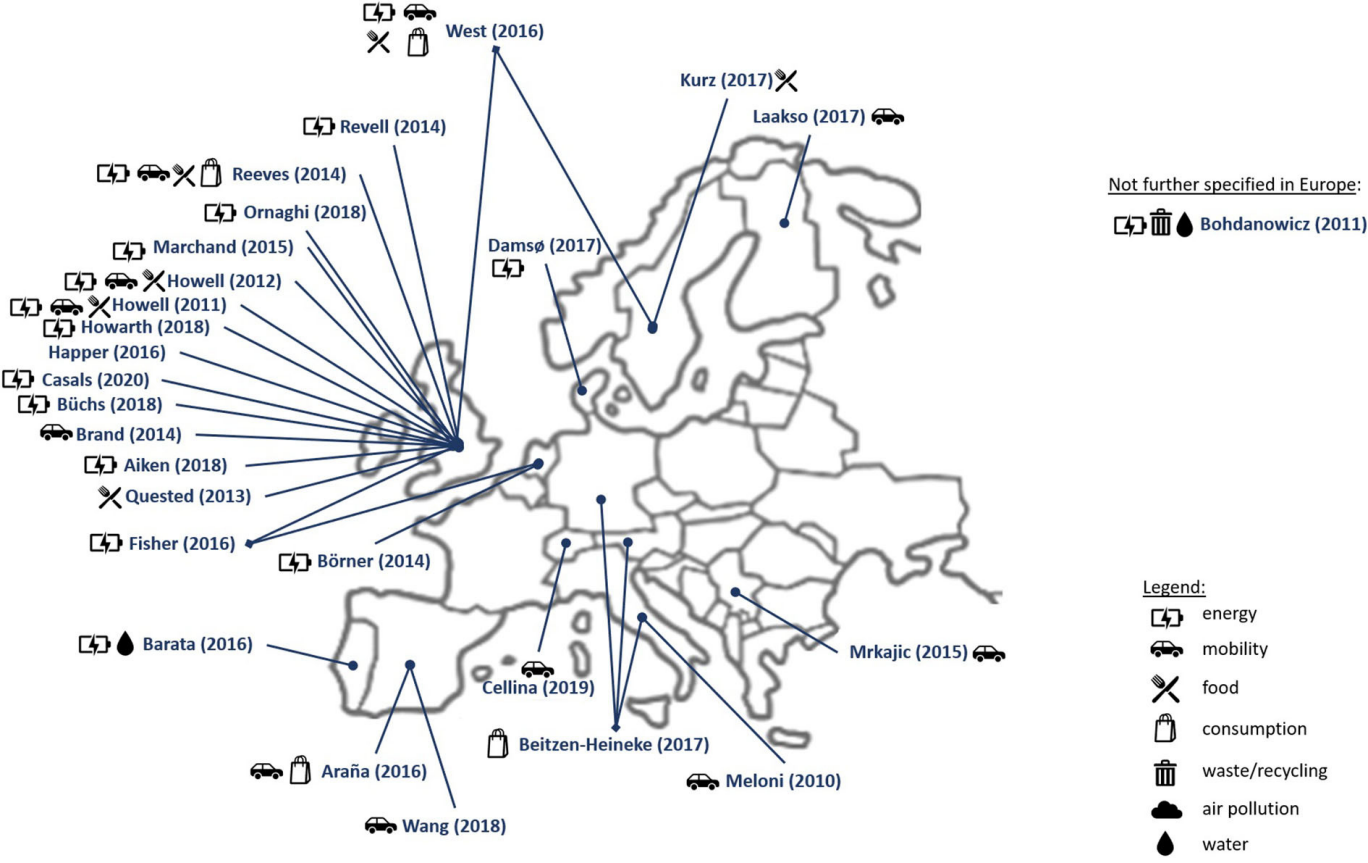
Graphical summary of the results regarding studies, which were only conducted in Europe, regarding location and sector, which the intervention studies aimed at (own illustration).

Figures 2, 3 illustrate the 48 studies that were conducted in Europe (with Austria *n* = 1, Denmark *n* = 5, Finland *n* = 1, Germany *n* = 1, Italy *n* = 2, Netherlands *n* = 5, Portugal *n* = 1, Serbia *n* = 1, Spain *n* = 2, Sweden *n* = 6, Switzerland *n* = 1, United Kingdom *n* = 21) with one study only stating “Continental Europe” without further specifying the location (Bohdanowicz et al., 2011; Howell, 2011, 2012; Meloni et al., 2011; Quested et al., 2013; Brand et al., 2014; Reeves et al., 2014; Revell, 2014; Börner et al., 2015; Marchand et al., 2015; Mrkajic et al., 2015; Araña and León, 2016; Fisher and Irvine, 2016; Happer and Philo, 2016; West et al., 2016; Barata et al., 2017; Beitzen-Heineke et al., 2017; Damsø et al., 2017; Laakso, 2017; Aiken, 2018; Büchs et al., 2018; Howarth and Roberts, 2018; Kurz, 2018; Ornaghi et al., 2018; Wang and Boggio-Marzet, 2018; Wynes et al., 2018; Cellina et al., 2019; Casals et al., 2020).

One review only stated OECD countries (Dawkins et al., 2019), and two reviews did not specify the countries from which the intervention studies originated (Kelly et al., 2013; Iweka et al., 2019). In summary, most intervention studies were conducted in the USA and Europe, especially in the UK, but developing as well as transition countries could also be included in this review.

According to the 54 publications included in this review, 74 studies focused solely on energy, 12 on mobility, 10 on food, two on consumption, two on waste/recycling, and one on air pollution. Seven studies focused on more than just one emitting sector: one study targeted mobility and consumption in the tourism sector; one energy and water; one energy, water, and waste; two looked at energy, food, and mobility; and two targeted energy, food, mobility, and consumption. Consequently, PEB interventions for all emitting sectors of an individual could be identified, with the majority focusing on energy. Interestingly, no studies targeting air travel were found, which is in concordance with previous reviews (Wynes et al., 2018).

The level at which the intervention took place, is divided into (1) policy, (2) community, or (3) individual. Six publications, including one review, targeted the policy, eight studies focused on the community, and the majority, 33 publications, targeted the individual level only. Two reviews and five intervention studies described activities involving both the individual and community level. Table 2 summarizes the results including the targeted sector and level, intervention type, and effectiveness.

According to the evidence pyramid, randomized controlled trials (RCT) that randomly assign participants to a control or intervention group are the most reliable source of evidence (Murad et al., 2016). However, only 13 studies had an RCT design (see Table 2) although most studies provided a comparison with a control group or baseline (pre-intervention) measure.

### Evidence-based effectiveness of single intervention types

Evidence from the literature endorsed Staddon et al. (2016) statement that interventions involving social influence, and/or structural measures seem to be particularly effective, and showed that feedback and policies also have the power to lead to significant emission reductions. Additionally, investing in behavior-changing PEB interventions can lead to a monetarily measurable return on investment: for example, Carrico and Riemer (2011) estimated that their interventions saved $15 (peer education) and $32 (feedback) in electricity costs for every dollar spent. However, the evidence-based effectiveness of the eight different intervention types mentioned in Section Pro-environmental behavior change interventions clearly shows that no intervention type on its own was very successful in promoting long-term behavior change. In summary, the studies demonstrated the following:

Providing information alone did not affect PEB, although it seemed to increase knowledge and change attitudes. However, this knowledge was not turned into PEB action, e.g., Showed that ambient learning displays had no significant influence on the conservation activities performed in the workplace (Hall et al., 2013; Börner et al., 2015; Morris et al., 2016; West et al., 2016; Barata et al., 2017; Büchs et al., 2018; Iweka et al., 2019). This is in line with existing literature by Stern and Abrahamse (Stern, 2000; Abrahamse, 2019). Interestingly, evidence showed that information alone can also backfire, i.e., lead to an increase in energy consumption (Carrico and Riemer, 2011; Staddon et al., 2016). Staddon et al. (2016) and Ornaghi et al. (2018) found that tailored information was more effective than general information. To increase this effect, information should be easy to understand and originate from a trusted source (Carrico and Riemer, 2011; Büchs et al., 2018).

Furthermore, Matsui et al. (2014) showed that providing feedback by using energy meters alone could not lead to a reduction in energy consumption, because the participants did not know how to reduce their consumption. Additionally, evidence showed that there is no difference in effectiveness between general feedback and feedback coming from a trusted source, e.g., peers (Carrico and Riemer, 2011).

According to Barata et al. (2017) commitments alone also do not affect PEB in the absence of other intervention types. Increased PEB could only be detected for commitment in combination with an educational intervention, but not without the other intervention.

Interestingly, evidence showed that nudging in terms of small changes to the environment had positive effects on its own regarding behavior change but only if the desired behavior and the needed skills, e.g., recycling, were already induced in individuals (Largo-Wight and Wight, 2013; Kurz, 2018).

Reeves et al. (2014) study even posed the question of whether social influence, e.g., in the form of community initiatives, can induce significant changes to their local area at all. Concerning incentives, Staddon et al. (2016) found that social rewards that are given publicly outperform other forms of incentives, e.g., money. Nevertheless, incentives (regardless of what kind and how much) alone do not promote behavior change—after an incentive is removed, individuals return to their old habits (Asensio and Delmas, 2015; Iweka et al., 2019). However, being rewarded for PEB can further enhance the behavior, e.g., payments for not using the company's parking spaces (Wynes et al., 2018).

Interventions solely using gamification showed small positive effects during the intervention (Ro et al., 2017; Casals et al., 2020). Furthermore, Ro et al. (2017) indicated that individuals who changed their behavior during the gamification intervention were likely to still show this behavior at 1-year follow-up. However, engaging individuals to partake in games and maintain long-term involvement was a crucial issue in all gamification interventions. Usually, individuals' engagement declined over time, thereby limiting long-term behavior change (Cellina et al., 2019; Casals et al., 2020).

Regarding policies and compliance with them, the kind of policy is important: if a policy is mandatory, e.g., laws or a municipality only supplying “green energy” to all consumers, pro-environmental measures are automatically implemented without (or with only a limited need) for behavior change; if a policy is on an individual, voluntary basis without being enforced, individuals have the choice to comply with the policy or not. Bohdanowicz et al. (2011) reported that a hotel chain's sustainability policy was used to engage employees in PEB activities. However, the employees' engagement declined over time, leading to new efforts to re-engage employees. According to Hoicka et al. (2014), Marchand et al. (2015), and Howarth and Roberts (2018) policy programs suggested that voluntary energy audits for homeowners, who receive suggestions for retrofitting to reduce energy consumption, had no effect at all when they were not combined with economic incentives. If the energy audit was free of charge and technical improvements, e.g., retrofitting, were reimbursed, individuals were very interested in the implementation of energy-reducing techniques. Consequently, the resulting energy reduction was also high. A similar policy including the installation of a set of free-of-charge retrofitting measures, if necessary, led to a lower but still positive result. If no free-of-charge installations were made, and the costs of retrofitting actions had to be paid by homeowners, e.g., *via* a loan, no behavioral change or significant energy reduction could be measured. Consequently, the different results seemed to be based on economic incentives alone with apparently no effect of the policy itself to significantly reducing consumption (Morris et al., 2016).

Lastly, while structural measures can result in emission reductions, they are not always associated with behavior change. For example, one-time installations can reduce energy consumption significantly, like Schultz et al. (2015) LED bulbs, but do not change an individual's behavior (Revell, 2014; Keall et al., 2015). According to Brand et al. (2014) additional infrastructure, e.g., for cycling or walking, without any other structural changes (roads closed or turned into one-way streets to make car use less convenient) did not change behavior toward switching from car use to environmentally-friendly alternatives. Instead, additional trips were made, mostly for recreational purposes, using the new infrastructure.

Consequently, the evidence shows that various intervention types should be combined instead of using only one single intervention type to change behavior. This is in concordance with findings from existing literature like Stern (Stern, 2000). For example, providing tailored information like health and risk information to persons with children or chronic diseases to raise awareness, and giving feedback on PEB actions afterward was shown to increase long-term commitment as well as PEB actions and significantly enhance behavior change (Hall et al., 2013; Asensio and Delmas, 2015; Morris et al., 2016; Staddon et al., 2016; Büchs et al., 2018; Iweka et al., 2019). The combinations of “feedback and educational/tailored information on how to change behavior” or “feedback and goal setting or commitment” (Iweka et al., 2019) are evidently able to promote behavior change (Matsui et al., 2014; Iweka et al., 2019). According to Wynes et al. (2018) most studies regarding energy used feedback (90% of all studies), and either paired it with information or gamification elements like rewards. Notably, only positive feedback should be given to avert negative responses from individuals (Carrico and Riemer, 2011; Staddon et al., 2016).

### Very effective interventions use combinations of intervention types or make the sustainable option the default

Fifteen publications described very effective interventions (see Table 2) to change behavior and enhance PEB activities by (a) reducing energy consumption (*N* = 13), (b) encouraging recycling (*N* = 2), or (c) reducing car use (*N* = 1). They incorporated improved (infra-)structures, education, feedback, and enablement as well as making the sustainable option the default.

Dams8 et al. described climate action plans with reduction targets, which were the basis for 51 mitigation initiatives using policies and structural measures. For example, the initiatives included improving the district's heating system, expanding wind power, or switching fuels in the municipalities' transport fleets (Damsø et al., 2017).

According to Hoicka et al. (2014) a government-initiated program encouraged individuals to invest in retrofitting and energy-reducing measures. After a free-of-charge home visit, recommendations were given to individuals, who could then decide if they wanted to retrofit their home and what measures to install. After improving their homes, they could get financial rewards.

Laakso (2017) gave an example of making the sustainable option the default. In her intervention, households had to sell one of their cars, which could be either their only or second car. In exchange, they received free travel cards for local (bus) services. This disruption of everyday routines led to new mobility behavior, which was mostly maintained at follow-up with only one household buying a new car after the intervention. As a result, this one-time decision resulted in a remarkable reduction in car-induced emissions.

Largo-Wight and Wight improved the infrastructure to make PEB more convenient by adding indoor opportunities to recycle cans and bottles. This “nudge” increased the recycling rate compared to the default mode, which only provided bins outside the buildings. Consequently, recycling behavior became more convenient for individuals (Largo-Wight and Wight, 2013).

Wynes et al. stated that individuals adopt “green energy” from their suppliers nearly 10 times more often (69.1%) if it is provided as the default with the possibility to opt-out than do individuals (7.2%) who have to actively purchase the “green energy” option. Additionally, Wynes et al. found that feedback effectively helped to reduce energy consumption (“[…] average 133 kgCO_2_e reductions over 17 interventions in 11 studies […]” (Wynes et al., 2018, p. 12), and nudging as well as making the sustainable option the default effectively reduced the consumption of meat [“[…] average of 144 kgCO_2_e reductions over three interventions in one study […]” (Wynes et al., 2018, p. 12).

According to Staddon et al., 21 of 22 interventions used education or information whereby information that is easy to understand, contextualized, and from a trusted source was most effective. No reviewed intervention incorporated training. Five of the eight most effective studies used technological solutions for automation, i.e., automatically turning off appliances or adjusting (heating) settings. While these automations do shift the emission-saving behavior to technology, individuals can easily override or reset the settings if they are not aligned with their personal preferences. Overall, 7 of the 12 best performing studies included enablement, i.e., individuals were in control of devices and changing settings in combination with information on PEB. Furthermore, social influences appeared to enhance PEB (Staddon et al., 2016).

Dowd et al. (2012) described how fact sheets and group sessions enabled individuals to reduce their emissions and even inspired them to lobby their government to create a sustainable future. For example, “[…] emissions from beef showed the greatest decline (30%), followed by household energy (23%), spending (21%), transport (16%), and waste (16%)” (Dowd et al., 2012, p. 268).

Fisher and Irvine reviewed four small-group interventions incl. information material focusing on energy reductions. All groups increased their PEB compared to the control group with PEB maintained and even increased 3 years after the intervention had ended (Fisher and Irvine, 2016).

Howell described results from Carbon Rationing Action Groups, whose participants live with yearly carbon allowances that should not be exceeded. Group members encourage each other to reduce their carbon footprint and exchange experiences. Most groups incorporated financial penalties for exceeding the carbon target, or implemented trading “allowances” with other members who were significantly lower than the target (Howell, 2012).

A government-initiated program rewarding municipalities for every resident who signed up for paying a surcharge of 50% or 100% of their energy consumption to finance the expansion of “green energy” is described by Jacobsen et al. (2013) municipalities had to pledge to purchase a share of their services' energy from “green energy sources.” If the number of residents who signed up exceeds a certain threshold, the municipality qualifies as “green” and receives free solar panels in proportion to the number of signed-up households. Especially the time around reaching the threshold is characterized by an increase in sign-ups, showing the social networking of individuals to reach a community goal, which led to a large number of “green energy” consumers.

Morris et al. (2016) described a program using home energy assessment incl. recommendations, for-free installations of measures, incentives, and quarterly energy efficiency information *via* electricity bills for island residents to reduce energy consumption. Additionally, reducing energy usage was a topic of discussion among islanders. As a result, after about 4 years the peak electricity demand had declined to below pre-intervention levels.

Educating individuals and training skills was described by Hammed et al. (2018) to reduce environmental pollution and increase recycling rates, a community-based intervention in Nigeria with community training was conducted to sensitize residents and develop as well as practice skills regarding waste management. None of the participants separated waste before the intervention whereas, after the intervention, 67.3% of the residents separated their waste. Additionally, harmful waste disposal practices were reduced, e.g., stream dumping decreased from 26.7 to 0.0%.

Giving feedback, setting goals, and engaging individuals *via* gamification led to the most significant reductions in energy consumption according to Iweka et al. (2019) review.

The Tokyo Cap-and-Trade Program to reduce emissions based on an emission target from non-residential buildings was described by Nishida et al. (2016) retrofitting measures focusing on heating/air conditioning and lighting, as well as social influences *via* organizational promoters led to high reductions. An additional factor for this significant sensitization and a significant decrease in energy consumption was the energy crisis following the earthquake and Fukushima nuclear reactor incident in March 2011.

These successful interventions used combinations of information or education and training to enable individuals in changing behavior, goal setting, and improved infrastructures or setting the sustainable option as a default. Additionally, the encouragement using social influences, e.g., group discussions or role models, is highlighted as part of effective interventions. This is in line with the previous findings of Abrahamse (2019): block leaders and social modeling are effective in encouraging behavior change with modest to large effects.

### Any intervention is better than none

According to the evidence, most interventions only showed small positive effects (see Table 2). As illustrated in Table 2, some interventions did not influence the targeted behavior at all. Nevertheless, small positive effects in non-targeted behavior could be achieved as described by Brand et al. (2014) although the infrastructural intervention was ineffective for its main outcome measure (CO_2_ emission in the mobility sector), it effectively promoted walking for recreation, i.e., it improved health-related behavior.

In summary, except for one household participating in an energy trial (Matsui et al., 2014), there were no negative impacts (i.e., increasing negative impact on the environment) resulting from the interventions. In fact, this one outlier resulted from changing individual circumstances during the intervention and not from the intervention itself (Matsui et al., 2014). This is in concordance with Chiu et al. (2020) results from comparing their interventions with “no intervention” as they found that any intervention is better than none.

To highlight common denominators in the studies, the following sections cluster evidence according to the factors found including anecdotal examples.

### Emotions, social, and cultural factors as evidence-based drivers and barriers to behavior change

Existing literature like Gifford (2011) name and discuss the psychological barriers to individual behavior change concerning PEB. For example, the impacts of individual behavior and the resulting GHG emissions on the environment are mostly distant or as Hargreaves found “invisible” and, thus, are perceived as “unrelated” to individuals' daily lives (Gifford, 2011; Hargreaves et al., 2013). Consequently, interventions need to raise awareness and make their objective relevant to participants to start the behavior change process. This means that there cannot be a “one size fits all” intervention approach. Instead, interventions need to be adaptable or customizable to individuals and their needs (Andersson et al., 2018). This includes the consideration of internal factors like emotions and external factors such as cultural factors, which is in line with the Model of PEB (Kollmuss and Agyeman, 2002; Araña and León, 2016; Benka-Coker et al., 2018).

For example, Benka-Coker et al. (2018) found that health and cost benefits alone are not motivating enough, if not “relevant,” to promote PEB alternatives when it is inconvenient or not in line with traditions. Benka-Coker et al. (2018) conducted two interventions providing less GHG-emitting and air-polluting ethanol cookstoves to replace firewood to (a) refugees in different refugee camps and (b) low-income urban households. Depending on the origin of the refugees, the cook stove was either found to be suitable for all cooking needs or could not entirely replace firewood for traditional stoves, because it was not in line with customs, e.g., using large traditional pots for porridge, preparing traditional meals, or having ceremonial coffee. Interestingly, the more resources and alternatives participants had, the less successful the program was: in the refugee camps the ethanol stove was used as the primary stove, whereas the low-income urban participants practiced so-called “stove stacking,” i.e., using two to five different kinds of stoves for different foods or purposes such as ceremonies.

Ruiz-Tagle and Schueftan showed that improving wood-burning technologies alone can lead to further problems because the user's behavior in operating the technology impacts the effectiveness of the improvement. For example, policies in Chile specified that only highly efficient “double combustion” stoves are to be available to the Chilean public. This led to decreased indoor but increased outdoor air pollution. Ruiz-Tagle and Schueftan used low-cost nudges in the form of information sign magnets to be installed above the stove's damper, aligning with the possible settings, to inform about the wood stove's emissions at the chosen setting. The damper setting regulates the airflow and, thus, the wood fuel combustion. Combustion and air quality improve with high air inflow, but the open flame might lead to higher firewood consumption and, consequently, increased wood-fuel expenses. Therefore, in Chilean households, the damper is mostly set to higher emitting settings with decreased air inflow. After field assistants provided information about the connection between damper settings and air quality including periodic check-in phone calls or visits, the individuals' behavior shifted to less polluting damper settings (Ruiz-Tagle and Schueftan, 2021).

However, Boso et al. (2019) demonstrated that awareness of a (health) problem, e.g., air quality, does not automatically engage individuals in behavior change. Instead, individual perceptions of the severity of the risk determine the willingness to participate in the intervention program.

On the other hand, Asensio and Delmas (2015) showed that persons with children provided with tailored, i.e., relevant, information about health issues like air pollution and asthma in children originating from energy consumption reduced their energy consumption by up to 19% compared to the controls. Other participants receiving the same information only achieved reductions of 8.2%. Happer and Philo highlighted that it is important to communicate risks (e.g., regarding health or property) in such a way that people can directly relate them to their experiences (Happer and Philo, 2016). This enhances the perceived relevancy in individuals.

Other examples of emotions and social factors as drivers and barriers are interventions regarding food, which is in line with existing literature like Stoll-Kleemann and Schmidt (2017). Behavior changes regarding food are not maintained long-term and interventions showed different levels of success depending on gender (Kelly et al., 2013). Besides monetary barriers to healthy eating, Kelly et al. (2013) concluded that nutritional interventions should include techniques to decrease emotional eating and increase social support. For example, men perceived certain behavior changes regarding diet as risking social ramifications and, thus, did not want to engage in such changes. According to the nudging intervention of Kurz, the sales of vegetarian dishes increased, but with varying numbers across different kinds of dishes (Kurz, 2018). Thus, vegetarian patties were more in-demand than stews, which could be related to their appearance resembling typical meat-related dishes like burgers (Kurz, 2018). Other interventions even showed backfiring effects with increased meat consumption due to the intervention: the authors assume that they unconsciously stimulated participants by repeatedly directing their attention to meat-related images and texts (Klöckner and Ofstad, 2017; Wynes et al., 2018).

Emotions also play a role in consumption, e.g., travel decisions. Araña and León found that CO_2_ emissions (measured as tons per person) increased when individuals were under time pressure or sad while booking a tourist package. Carbon taxes, CO_2_ labeling, and a high level of empathy for future generations led to decreasing CO_2_ emissions with high taxes (>10%) and empathy being statistically significant and, thus, most efficient. Consequently, interventions may unintentionally discourage PEB activities, if they cause sadness in people (Araña and León, 2016).

Additionally, Mrkajic et al. (2015) promoted emission-reduced mobility, i.e., cycling, by providing a bicycle parking area that students perceived as secure. Previous bicycle parking areas were perceived as unsafe and, thus, presented a barrier to changing mobility behavior.

Marchand et al. (2015, p. 104) conclude that individual choices and adapting interventions to align with an individual's existing routines, could “[…] prevent rejection of potentially beneficial measures [...].” For example, the convenience of recycling opportunities influences the impact of encouraging recycling, and wall insulation measures affect the appearance and/or size of a house (Carrico and Riemer, 2011; Marchand et al., 2015). Revell stated that installed retrofitting measures might even be removed by individuals who perceive them as inconvenient or ineffective (Revell, 2014).

In summary, when individuals have alternatives, they will use the most convenient option, e.g., motorized travel (see Brand et al., 2014), stick to their former behavior, e.g., cooking behaviors (see Benka-Coker et al., 2018), or use the option that makes them feel better (see Araña and León, 2016). Especially traditions or socio-cultural expectations are relevant for certain sectors like food and mobility. For example, this is due to traditional eating habits, a greater spatial spread of family and work environments, or the greater array of opportunities for low-cost travel by car and plane (Benka-Coker et al., 2018; Büchs et al., 2018). Additionally, external barriers (infrastructural and social) and “[…] concerns about impracticality, inconvenience, unreliability and higher costs […]” (Büchs et al., 2018, p. 290) reduce the willingness to change behavior.

### Enabling and motivating behavior change based on evidence

To engage and motivate individuals in behavior change, PEB-related goals should be both achievable and challenging. “Achievable” means that individuals need to be able to change, i.e., have the knowledge of alternatives and means to alter behavior as demonstrated by Matsui et al. (2014). Fisher and Irvine found that the simpler the behavior change advice the more reductions can be achieved (Fisher and Irvine, 2016). For the energy sector, Asensio and Delmas found that information on appliance-level electricity consumption was valued as most useful, surprising individuals by reflecting on how much or little appliances consumed energy, or were being used (Asensio and Delmas, 2015). This led to “[…] primarily […] plug load and lighting behavioral changes” (Asensio and Delmas, 2015, p. 4) with “[…] the most commonly reported behavioral changes […] [being] turning off unused lights, unplugging electronics, and charging devices when not in use” (Asensio and Delmas, 2015, p. 5). Additionally, Jorgensen et al. (2021) found that after giving participants the information about reducing energy consumption during peak demand periods, the 8-h notification of an upcoming energy peak demand period was more effective than 24-h notifications with a “2-h before peak demand period”-reminder. This result was unexpected due to the greater temporal distance and the authors speculated that the 24+2 scenario “[…] provoked psychological reactance” and, therefore, was experienced negatively by the participants (Jorgensen et al., 2021, p. 12).

According to Staddon et al. (2016) review, 7 of the 12 most effective studies used enablement (e.g., direct support, techniques, tools, and aids) to provide individuals with the necessary means to change their behavior—in addition to other intervention types. Quested et al. (2013) described how habitual elements of intervention activities using repetition, and linking related issues raised awareness and led to measurable positive effects. Furthermore, education in the form of active training showed positive effects on behavior change. For example, Hammed et al. (2018) showed this to be true for workshops regarding recycling management. Additionally, Wang and Boggio-Marzet (2018) demonstrated that a one-time training course regarding eco-driving immediately affected individuals' driving behaviors, e.g., leading to altered average/maximum speed, less aggressive acceleration, and measurable reductions in fuel consumption. However, the intervention did not include a follow-up and, thus, limits the findings to short-term behavior change only (Wang and Boggio-Marzet, 2018).

According to Reeves et al. (2014) and Cellina et al. (2019) long-term commitment to PEB activities, a prerequisite for behavior change, is problematic. Cellina et al. (2019) used commitments and social support to motivate long-term behavior change. Interestingly, “private commitments lead to higher energy conservation than public commitments” (Iweka et al., 2019, p. 5). In general, social influence and (peer) competitions seem to be very motivating in promoting PEB (Sintov et al., 2016; Staddon et al., 2016; Cellina et al., 2019). However, social influence needs persons to act as role models, e.g., “block leaders.” This can be achieved by peers, e.g., in Aiken's study neighbors, who share interests in certain sustainability options being brought together to share their thoughts or experiences, and thereby support and reinforce each other's behavior changes (Aiken, 2018). However, as Reeves et al. (2014) stated “[…] social movement framed around sustainability or climate change is likely to attract only limited levels of support and active participation [...]” (Reeves et al., 2014, p. 127). Long-term social influences, e.g., in the form of grassroots initiatives dissolve when no willing group or block leaders can be found.

### Low-cost behaviors and transition phases constitute good starting points for PEB interventions

According to the findings of Büchs et al. “[…] participants were mostly willing to consider changes which would either bring them a personal benefit (e.g., getting fitter by walking more) or which would not affect their lifestyles too much” (Büchs et al., 2018, p. 290) with varying degrees of willingness “[…] depending on what aspect of their lifestyle [the change] impacts” according to West et al. (2019, p. 404). Therefore, behavior change is mostly in favor of low-cost behavior, which is in line with Diekmann and Preisendörfer (2003) theory of low- and high-cost situations. For example, Barata et al. (2017) showed that students could more easily be motivated to adopt water-saving behavior than energy-saving behavior. Also, the behavior changes to switching off appliances when not in use reported by Cornelius et al. (2014) and Asensio and Delmas (2015) studies are easy to implement and, thus, low-cost behaviors.

Additionally, Hall et al. (2013), Quested et al. (2013), and Laakso (2017) state that the best opportunity to implement behavior change is during transition phases like moving to new accommodation, changing jobs, having a child, or joining/leaving the labor force. Such transitions already disrupt behavior patterns, making a change toward PEB and implementing new routines easier.

For example, Malan et al. (2020) found that a “Foodprint seminar” regarding food systems and sustainability aspects as a university course shifted students' dietary intake with a tendency to decrease their food-related carbon footprint, especially among frequent ruminant meat consumers. The seminar topics helped to motivate behavior change and increase food literacy by highlighting impacts on social justice and environmental sustainability. Since college and university are natural transition periods, the seminar's timing might also contribute to the described behavior change.

### The role of governments and politics

According to Dowd et al. (2012), Happer and Philo (2016), Damsø et al. (2017), and Dawkins et al. (2019), framework conditions set by national/local government influence how problems, e.g., GHG reductions, are tackled—especially when it comes to long-term effects. Governments can target and engage different sectors and groups. For example, if regulations specify that only the sustainable option, e.g., renewable energy, is to be used as a default, every individual, business, and sector will automatically consume more sustainable energy—in a few cases even without the need for active behavior change of individuals. Governments can also take a leading role, prioritize interventions, and provide funding, which is a frequently mentioned barrier regarding interventions, their resources (e.g., staff, and technology), and their scalability (Ruiz-Tagle and Schueftan, 2021).

For example, the government in Chile banned the use of wood-burning stoves in areas with high air pollution during emergency periods. Boso et al. (2019) described a governmental program to subsidize all costs (incl. a new stove, installation, and removal of the old stove) for replacing wood-burning stoves to support the transition toward more sustainable stove types.

However, this policy shifted indoor air pollution to outdoor air pollution because of the stove users' behavior, thereby calling for subsequent behavior change interventions (Ruiz-Tagle and Schueftan, 2021).

Another example is the Tokyo Cap-and-Trade Program to reduce emissions from non-residential buildings as described by Nishida et al. (2016) which led to high reductions. Policies to reduce energy consumption in non-residential buildings (e.g., offices, communication businesses, and data centers) can lead to significant emission reductions.

The reviewed studies also provide recommendations for policy-makers. Wang and Boggio-Marzet (2018) recommend that local policymakers and transport planners implement a so-called “green wave.” Coordinating traffic lights on routes with intensive traffic flows ensures constant speed and, thus, eco-driving with reduced ac-/decelerations. Additionally, mandatory eco-driving education should be integrated into driving license courses.

Furthermore, problematic issues can be addressed by using evidence. Since driving cars is the most dominant transport mode as described by Meloni et al. (2011) and Keall et al. (2015) policy-makers could subsidize more (infra)structural measures to encourage active travel modes, e.g., cycling.

Beitzen-Heineke et al. (2017) described the alternative retail concept of zero packaging grocery stores to reduce packaging waste, potential food waste due to packaging guidelines, or marketing (e.g., excessively large packaging). Individuals supporting the growing number of such stores despite the inconvenience resulting from a more time-consuming shopping experience, limited product range, and the need to clean and bring their own containers could provide a lobby for political packaging-related issues.

Consequently, individuals can influence their local government by lobbying and using their votes (Dowd et al., 2012; Happer and Philo, 2016; Damsø et al., 2017; Dawkins et al., 2019). However, one barrier to lobbying might be individuals' distrust of policy-makers as described by Happer and Philo (Happer and Philo, 2016).

## Discussion

All but 10 of the reviewed intervention studies took place in so-called developed, high-emitting countries, which need to reduce emissions most urgently. However, meeting social needs and emission goals, while enabling economic growth in developing and transition countries should also be brought into the focus of sustainability research.

In line with the findings from Wynes et al. (2018), this review identified more interventions targeting the energy sector than mobility, food, consumption, and waste/recycling combined. Most studies focused on individuals and changing their behavior.

### Limitations regarding the search strategy

The presented search strategy defined exemplary emission sectors of interventions by search words, but should not be limited to specific sectors. Therefore, publications concerning other emission sectors that appeared in the search were also included in this review. Since the search words made the search and its results reproducible, this approach should not undermine the systematic character of this review. Despite this broad approach, this review may be influenced by publication bias, thereby limiting its overall validity to the results found.

### Study designs and intervention types

This review's results are based on findings from the literature concerning behavior change interventions toward PEB and, consequently, the quality of these intervention studies. The study designs allow for biases compromising the quality of the results. For example, self-reported outcomes in questionnaires are vulnerable to biases such as “social desirability,” “recall,” and “reporting” (Meloni et al., 2011; Dowd et al., 2012; Revell, 2014; Sintov et al., 2016; Staddon et al., 2016; Ro et al., 2017; Iweka et al., 2019). Additionally, an individual's behavior might change simply by taking part in an intervention study (Hawthorne effect) (Iweka et al., 2019; Ruiz-Tagle and Schueftan, 2021). Furthermore, RCTs are not common in research regarding PEB-promoting interventions but would yield the most robust evidence. Interestingly, studies with an RCT design showed no or only small positive effects with only one successful outlier, namely a subgroup of people with children, who responded to health messages with higher reductions in energy use (Asensio and Delmas, 2015). This could suggest that behavior change toward PEB is not possible in all individuals.

The lack of success in the majority of interventions is often explained by a recruiting bias in voluntary sustainability interventions or, as Howell called it, “preaching to the converted” (Howell, 2011, p. 184). This means that individuals participating in the interventions cannot achieve high reductions or effects, because they have already adjusted their lifestyle toward more sustainability. Cellina et al. (2019) stated that the lack of an intervention effect could be due to the fact that the study participants in Zurich already showed more PEB than the average population. Büchs et al. (2018) also reported a PEB response bias and thus limited possible effects of the intervention. Like most researchers, Jacobsen et al. (2013) found that the majority of their participants had a higher level of education than the average population. Concerning her successful intervention, Laakso declared that “Many of the participants had considered giving up ownership of their cars already before the experiment, and seeing the announcement in a local newspaper was the incentive they needed to make the final decision” (Laakso, 2017, p. 138). Bohdanowicz also stated that the respondents of the survey are most likely more interested in environmental issues than non-responders (Bohdanowicz et al., 2011).

Since most studies are characterized by small sample sizes, as well as short interventions and, if applicable, follow-up periods, the effects, statistical results, and generalizability can be negatively influenced (Sintov et al., 2016; Iweka et al., 2019). For example, Barata et al. (2017) based their results on a 1-month intervention period aimed at teenagers, which are only a part of a given household, and a control sample for saving readings of *N* = 7. Chiu et al. (2020) had a study period of 4 weeks with 3 weeks of intervention and, thus, without the possibility to measure long-term behavior change. Cornelius et al. (2014) and Sintov et al. (2016) studies lasted 7 weeks without follow-up, Largo-Wight and Wight analyzed 8 weeks, with only a 4-week-long intervention, and Wang et al. conducted a 1-month-long study (Largo-Wight and Wight, 2013; Wang and Boggio-Marzet, 2018). The study by Meloni et al. (2011) was the shortest at 2 weeks, including a 1-week-long intervention.

Especially, interventions regarding the food sector would benefit from longer intervention durations and follow-up periods. Wynes et al.'s findings that “[…] the majority of studies described measurements for a period of weeks only, making it difficult to assess the persistence of any interventions” (Wynes et al., 2018, p. 15) are in concordance with the findings of this review. Especially, interventions in canteens, which mostly target one meal per work day and, consequently, five meals a week, need long intervention durations to create a robust effect. Only Malan et al. (2020) had a longer study period of one academic term, i.e., 10 weeks.

For food interventions aiming at increasing the sale of vegetarian dishes, e.g., in canteens, Kurz suggested examining a dish's food components in detail, because vegetarian food can cause higher GHG emissions than a meat dish. For example, this is true for cheese when used to substitute for meat, if the alternative is a dish with more climate-friendly meats such as poultry. Therefore, food interventions should consider measuring the sale of more vegan alternatives or dishes with fewer dairy products. Consequently, the results from sale-based interventions should be treated with caution (Kurz, 2018).

Furthermore, the setting and timing of interventions could bias results. For example, Carrico and Riemer collected baseline energy consumption data in summer (May to August), but the intervention and its data collection took place in fall/winter (September to December) when energy consumption naturally rises due to the use of heating (Carrico and Riemer, 2011). Meloni et al. (2011) conducted their first survey week in summer, while the intervention week was in autumn, which could also lead to measurement biases based on changed weather conditions. Cellina et al. (2019) stated that their mobility intervention might have been unsuccessful because it was conducted during winter, which—due to weather conditions—is less bike- and slow-mobility-friendly. Jorgensen et al. (2021) also stated that their measurements might be biased because students might just have left their residence during energy peak demand periods to use energy elsewhere on the campus.

Lasting reductions due to social influence, i.e., group interventions, are in line with Abrahamse et al. (2005), Abrahamse (2019) findings. According to Hall et al. (2013) face-to-face group interventions resulted in more actions performed as PEB changes than in online groups. However, Fisher and Irvine found that smaller groups will most likely consist of individuals with existing PEB intentions and, thus, limit activities on a wider societal scale (Fisher and Irvine, 2016).

Additionally, social influence *via* comparative feedback, e.g., using information about peers as a benchmark, could lead to a certain sense of competition and increased PEB, but it can also lead to non-compliance when individuals justify their behavior by using this benchmark. For example, if peers show less PEB it might serve as a demotivation to engage in further PEB activities since one's outcome is already better than the results of others. Additionally, distinctiveness (e.g., income, lifestyle, household characteristics) could be used as a justification for not engaging in further PEB activities or even increasing consumption (Wynes et al., 2018; Iweka et al., 2019).

### Behavior change models and intervention measurements

While some interventions did not consider Behavior Change Models, others most often mentioned the Theory of Planned Behavior (TPB), and the Transtheoretical Model (TTM). For example, Cellina et al. (2019) designed their intervention based on the TTM with different intervention types for each behavior change stage. The evidence showed that behavior change is influenced by multiple factors, e.g., emotions, and social and cultural factors, which supports Kollmuss and Agyeman's model of pro-environmental behavior (Kollmuss and Agyeman, 2002).

In concordance with Happer and Philo, the lack of knowledge and understanding regarding climate change and possible (individual) solutions were consistent across studies, which was measurably increased by interventions (Happer and Philo, 2016). This also supports the model of Kollmuss and Agyeman, who base environmental consciousness on knowledge, which influences emotions as well as values and attitudes (Kollmuss and Agyeman, 2002).

Another aspect supporting the model of pro-environmental behavior is that, according to the evidence, only combinations of interventions were really successful. Since the model includes barriers to every aspect of behavior, behavior change is more likely when various barriers are targeted and removed. A combination of interventions automatically addresses different factors, e.g., information targets knowledge, increasing awareness, and environmental consciousness while gamification, such as challenges, motivates individuals to engage in PEB.

The success of interventions is measured with different metrics, e.g., kWh, or kgCO_2_, and *via* various techniques, e.g., smart meter readings, GPS travel information, or electricity bills. Especially if interventions during different seasons are included, a control for changes in weather conditions would be advisable. However, since not all studies included such data, the comparability of findings is subsequently reduced.

Another aspect is that changes in emissions could originate from other sources, e.g., national changes/activities, which are outside of the intervention's scope. Additionally, with changing societies and advances in technology, e.g., more efficient appliances, the impact of an intervention is likely to change over time. All these aspects should be considered when assessing and comparing an intervention's effectiveness.

### The possibility for behavioral change

Although structural measures and automation are successful in reducing emissions, the question remains whether such measures can be called “PEB.” Technological solutions mostly do not aim to promote behavior changes. In fact, most one-time changes like sustainable default settings or switching to the “green” energy tariff can persist without maintenance and, thus, without the need for any behavior change, thereby preventing the risk of relapses or rebounds (Morris et al., 2016). However, Staddon et al. (2016) state that technological changes can create a new environment in which behavior change can be induced. For example, individuals who had retrofitting measures installed can serve as role models and examples to their social network when showing off the changes (Marchand et al., 2015).

However, large-scale behavior change toward PEB currently seems to be an unsolved challenge. Revell stated that the result of behavior change regarding energy and water savings behavior after home energy audit visits was negligible (Revell, 2014). According to Happer and Philo, the “[…] majority had not made changes to their behavior. […]” (Happer and Philo, 2016, p. 145) which suggests that behavior change is only possible in individuals who were willing to change prior to the intervention.

### Evidence-based recommendations for interventions on enhancing PEB

While deriving recommendations from evidence is considered best practice in most research areas, generalizability depends on the type, amount, quality, and consistency of the evidence. The most robust recommendations are based on multiple, independent studies of high quality. To indicate how robust a recommendation is, the terms “limited,” “medium,” or “robust” are used in the following.

The following recommendations can be derived from the evidence on how interventions for enhancing PEB should be designed and implemented:

First, make the sustainable option the default, or more convenient. Robust evidence shows that the fewer alternatives there are, the higher the level of compliance (Largo-Wight and Wight, 2013; Hoicka et al., 2014; Staddon et al., 2016; Damsø et al., 2017; Laakso, 2017).

Second, concentrate on high-impact behavior. Wynes et al. (2018) quantified how much emissions were reduced by interventions focused on individuals' behavior. As a result, the impact of behavior change can be quantified whereby interventions that focus on reducing car usage and the resulting emissions seemed to have the highest impact. Reducing household energy consumption had the second-highest impact followed by emission reductions regarding food. The robustness of this recommendation can be considered “medium” based on multiple data presented in reviews (Bundesministerium für Umwelt, 2018).

Third, interventions conducted in developing and transition countries prove that without previous sensitization even relatively low-effort interventions like education and training, e.g., on recycling, can have a high impact on behavior change toward PEB activities and environmental issues involving water, land, and air pollution. Since the publication basis for developing and transition countries is very limited, this finding should also be considered “limited” (Hammed et al., 2018; Ruiz-Tagle and Schueftan, 2021).

Fourth, as the most successful interventions prove and, thus, give robust evidence, use a combination of intervention types, e.g., tailored information to raise awareness and trigger engagement, education, and training to enable and self-empower individuals to change their behavior, and continuous feedback to enhance long-term commitment. Consequently, PEB change goals need to be achievable and challenging to engage and motivate individuals.

Fifth, disrupt routines or use transition phases to successfully change behavior (Hall et al., 2013; Quested et al., 2013; Laakso, 2017). Although only a limited number of interventions used this method and, thus, this recommendation would only be considered “medium,” further research and intervention planning should consider disrupting routines as it is known to increase possibilities for behavior change (Stieß and Rubik, 2015).

## Conclusions

This systematic literature review assessed the evidence-based effectiveness of 54 publications regarding behavior change interventions for enhancing PEB in individuals. The review identified very successful interventions to increase PEB, which were described in the results section. To the best of our knowledge, this paper is the first to conduct a review regarding the contents of behavior change interventions regardless of the emitting sector or targeted level and, thus, its findings advance the body of knowledge to plan and implement behavioral intervention studies.

In summary, the various interventions and intervention types to promote PEB differ in their effectiveness, leading to several evidence-based insights. No single intervention type by itself was successful in long-term behavior change and, according to the evidence, most interventions showed only small positive effects. Only 15 publications described very successful interventions. However, every intervention is better than none. This proves that effective interventions to increase PEB exist, but a combination of intervention types is the key to success. To change behavior, PEB goals should be both achievable and challenging to engage and motivate individuals. Additionally, emotions and social and cultural factors are both drivers and barriers to behavior change, in favor of the model of pro-environmental behavior designed by Kollmuss and Agyeman (Kollmuss and Agyeman, 2002). Interventions are most successful when the sustainable option is the default/new “normal,” or more convenient. Furthermore, the best opportunity to implement behavior change is during transition phases or after disrupting routines. For example, some authors conclude that interventions in college or university settings are promising. Moreover, interventions should concentrate on high-impact behavior, i.e., reducing (1) air travel and car usage, (2) household energy consumption, and (3) emissions regarding food. Interestingly, even relatively low-effort interventions in developing and transition countries can successfully change behavior toward PEB and reduce pollution of water, land, and air. Consequently, PEB interventions should include such countries more. Therefore, this paper contributes more generally to the growing literature on the broader topic of “PEB interventions” and in helping to develop very successful evidence-based interventions.

Future interventions should include follow-ups to monitor whether a changed behavior could be integrated as a habit in an individual's way of life and, thus, is maintained long after the intervention ended. Moreover, new interventions should have longer intervention or study durations as well as randomized controlled study designs. Additionally, research should focus on high-/low-impact as well as high-/low-cost behavior to develop interventions, which are more focused on high-impact but low-cost behavior changes, or avoid low-impact but high-cost behavior changes.

## Data availability statement

The original contributions presented in the study are included in the article/Supplementary material, further inquiries can be directed to the corresponding author/s.

## Author contributions

HR and SS-K: conceptualization and methodology and conducting the review. HR and SN: writing—original draft preparation. HR, SN, and SS-K: writing—review and editing. All authors have read and agreed to the published version of the manuscript.

## Conflict of interest

The authors declare that the research was conducted in the absence of any commercial or financial relationships that could be construed as a potential conflict of interest.

## Publisher's note

All claims expressed in this article are solely those of the authors and do not necessarily represent those of their affiliated organizations, or those of the publisher, the editors and the reviewers. Any product that may be evaluated in this article, or claim that may be made by its manufacturer, is not guaranteed or endorsed by the publisher.

## Supplementary material

Supplementary Data Sheet 1 Database searches.

Supplementary Data Sheet 2 Excluded publications.
