# Supplementary material for: A systematic review to assess the evidence-based effectiveness, content, and success factors of behavior change interventions for enhancing pro-environmental behavior in individuals
Source: Front Psychol. 2022 Sep 5;13:901927. doi: 10.3389/fpsyg.2022.901927 (PMC9486705; doi:10.3389/fpsyg.2022.901927)
Supplement: Supplementary Data Sheet 1 — Database searches. [file Data_Sheet_1.docx]

Supplementary Material – Database Searches

All database searches were conducted on April 1^st^, 2020.

Supplement - Table 1: Database Search Strategy and Results using Embase (including MEDLINE)

| **ID** | **Search** | **Results** | **Description** | **Timestamp** |
| --- | --- | --- | --- | --- |
| #1 | A | 386,729 | Selection: All Fields | 09:32:45 |
| #2 | B | 5,183,023 | Selection: All Fields | 09:33:59 |
| #3 | C | 1,783,423 | Selection: All Fields | 09:34:57 |
| #4 | D | 788,902 | Selection: All Fields | 09:35:46 |
| #5 | E | 138,625 | Selection: All Fields | 09:36:39 |
| #6 | #1 AND #2 | 62,416 | Selection: All Fields | 09:37:28 |
| #7 | #6 AND #3 | 9,052 | Selection: All Fields | 09:38:14 |
| #8 | #7 AND #4 | 463 | Selection: All Fields | 09:38:34 |
| #9 | #8 AND #5 | 170 | Selection: All Fields | 09:39:01 |
| #10 | #9 AND ([english]/lim OR [german]/lim) | 170 | Selection: Languages | 09:39:29 |
| #11 | #10 AND [2010-2020]/py | 162 | Selection: Publication Years from | 09:39:52 |

Supplement - Table 2: Database Search Strategy and Results using APA PsycInfo, APA PsycArticles and Psychology and Behavioral Sciences Collection via EBSCOhost

| **ID** | **Search** | **Results** | **Description** | **Timestamp** |
| --- | --- | --- | --- | --- |
| #1 | A | 4,528,458 | Selection: All Fields | 08:17:45 |
| #2 | B | 845,655 | Selection: All Fields | 08:19:59 |
| #3 | C | 97,396 | Selection: All Fields | 08:20:46 |
| #4 | D | 447,119 | Selection: All Fields | 08:23:05 |
| #5 | E | 8,864 | Selection: All Fields | 08:24:39 |
| #6 | #1 AND #2 | 827,144 | Selection: All Fields | 08:25:48 |
| #7 | #6 AND #3 | 20,284 | Selection: All Fields | 08:26:34 |
| #8 | #7 AND #4 | 3,547 | Selection: All Fields | 08:27:11 |
| #9 | #8 AND #5 | 130 | Selection: All Fields | 08:27:31 |
| #10 | #9 AND (Englisch OR English OR Deutsch OR German) | 129 | Selection: Language | 08:28:17 |
| #11 | #10 AND (2010-2020) | 103 | Selection: Publication Year | 08:29:51 |
| #12 | #11 AND Peer reviewed | 79 | Selection: tick box „peer reviewed” | 08:30:13 |

Supplement - Table 3: Database Search Strategy and Results using Web of Science Core Collection

| **ID** | **Search** | **Results** | **Description** | **Timestamp** |
| --- | --- | --- | --- | --- |
| #1 | A | 1,007,479 | Selection: All Fields | 09:59:35 |
| #2 | B | 6,855,898 | Selection: All Fields | 10:00:59 |
| #3 | C | 5,463,504 | Selection: All Fields | 10:02:46 |
| #4 | D | 1,672,561 | Selection: All Fields | 10:04:50 |
| #5 | E | 812,173 | Selection: All Fields | 10:05:39 |
| #6 | #1 AND #2 | 207,525 | Selection: All Fields | 10:08:48 |
| #7 | #8 AND #3 | 88,158 | Selection: All Fields | 10:09:34 |
| #8 | #9 AND #4 | 8,830 | Selection: All Fields | 10:10:11 |
| #9 | #10 AND #5 | 3,425 | Selection: All Fields | 10:10:31 |
| #10 | 2010/01/01 to present (2020/03/20) | 3,150 | Selection: Date - Publication | 10:07:51 |
| #11 | Englisch OR English OR Deutsch OR German | 3,134 | Selection: Language | 10:07:17 |
| #12 | NOT Proceedings paper, editorial material | 2,933 | Selection: NOT Proceedings paper, editorial material | 10:07:32 |
