# Supplementary material for: A systematic review to assess the evidence-based effectiveness, content, and success factors of behavior change interventions for enhancing pro-environmental behavior in individuals
Source: Front Psychol. 2022 Sep 5;13:901927. doi: 10.3389/fpsyg.2022.901927 (PMC9486705; doi:10.3389/fpsyg.2022.901927)
Supplement: Supplementary Data Sheet 2 — Excluded publications. [file Data_Sheet_2.docx]

Supplementary Material – Excluded publications

Supplement - Table 4: Excluded publications

| **author, title, reference** | **reason for exclusion** |
| --- | --- |
| Achtnicht, M. German car buyers’ willingness to pay to reduce CO2 emissions. Climatic Change 113, 679–697 (2012). https://doi.org/10.1007/s10584-011-0362-8 | Does not meet inclusion criteria: modell only, no PEB intervention |
| Ahamer, G. "Can Educational Approaches Help to Revolutionize Quantitative Solutions for Climate Change?." In Revolutionizing Education through Web-Based Instruction. edited by Mahesh Raisinghani, 1-19. Hershey, PA: IGI Global (2016).  http://doi:10.4018/978-1-4666-9932-8.ch001 | Does not meet inclusion criteria: no PEB intervention |
| Ahmad, S.; Puppim de Oliveira, J. A. “Determinants of urban mobility in India: Lessons for promoting sustainable and inclusive urban transportation in developing countries”. Transport Policy, Volume 50, 106-114 (2016). https://doi.org/10.1016/j.tranpol.2016.04.014 | Does not meet inclusion criteria: modell only, no PEB intervention |
| Ahvenniemi, H.; Häkkinen, T. "Households’ potential to decrease their environmental impacts: A cost-efficiency analysis of carbon saving measures". International Journal of Energy Sector Management, Vol. 14 No. 1, 193-212 (2020). https://doi.org/10.1108/IJESM-02-2019-0009 | Full-text not available to review’s authors |
| Ajayi-Banji, A. et al. "Deleterious Emission Abatement through Structured Energy Use Pattern: A North Central Nigeria Perspective". Environmental and Climate Technologies 17, 1, 76-90 (2016). https://doi.org/10.1515/rtuect-2016-0007 | Does not meet inclusion criteria: Life Cycle Assessment Modell only, no PEB intervention |
| Alberini, A.; Bigano, A. “How effective are energy-efficiency incentive programs? Evidence from Italian homeowners”. Energy Economics, Volume 52, Supplement 1, S76-S85 (2015). https://doi.org/10.1016/j.eneco.2015.08.021 | Does not meet inclusion criteria: modell based on questionnaire only, no PEB intervention |
| Alcock, I. et al.“‘Green’ on the ground but not in the air: Pro-environmental attitudes are related to household behaviours but not discretionary air travel”. Global Environmental Change, Volume 42, 136-147 (2017). https://doi.org/10.1016/j.gloenvcha.2016.11.005 | Does not meet inclusion criteria: online framing experiment to measure preferences only, no PEB intervention |
| Al Sabbagh M. Social Learning and the Mitigation of Transport CO2 Emissions. Climate.5(1):6, 2017. https://doi.org/10.3390/cli5010006 | Does not meet inclusion criteria: measures preferences only, no comprehensible indication or methodology indicating that this is an PEB intervention |
| Amelung, D. et al. „Human health as a motivator for climate change mitigation: results from four European high-income countries”. Global Environmental Change, Volume 57, 101918 (2019). https://doi.org/10.1016/j.gloenvcha.2019.05.002 | Does not meet inclusion criteria: measures intention-based willingness based on questionnaire only; no PEB intervention |
| Anderson, B.; Bernauer, T. “How much carbon offsetting and where? Implications of efficiency, effectiveness, and ethicality considerations for public opinion formation”. Energy Policy, Volume 94, 387-395 (2016). https://doi.org/10.1016/j.enpol.2016.04.016 | Does not meet inclusion criteria: online framing experiment to measure preferences only, no PEB intervention |
| Andersson, A.; Winslott Hiselius, L.; Adell, E. “Promoting sustainable travel behaviour through the use of smartphone applications: A review and development of a conceptual model”. Travel Behaviour and Society, Volume 11, 52-61 (2018).  https://doi.org/10.1016/j.tbs.2017.12.008. | Does not meet inclusion criteria: description of methods not detailed enough – unclear when and in which databases search was conducted; not every study’s methodology (e.g. number of participants, design) was described |
| Asvatourian, V. et al. “Relationship between pro-environmental attitudes and behaviour and dietary intake patterns”. Sustainable Production and Consumption, Volume 16, 216-226 (2018). https://doi.org/10.1016/j.spc.2018.08.009 | Does not meet inclusion criteria: questionnaire only; no PEB intervention |
| Aung, T.W. et al. “Health and Climate-Relevant Pollutant Concentrations from a Carbon-Finance Approved Cookstove Intervention in Rural India”. Environ Sci Technol. 2016 Jul 5;50(13):7228-38. https://doi.org/10.1021/acs.est.5b06208 | Full-text not available to review’s authors |
| Babakhani, N.; Ritchie, B.W.; Dolnicar, S. “Improving carbonoffsetting appeals in online airplane ticket purchasing: testing new messages, and using new testmethods”. Journal of Sustainable Tourism, 25:7, 955-969 (2017). https://doi.org/10.1080/09669582.2016.1257013 | Does not meet inclusion criteria: laboratory experiment of messages, no PEB intervention but stimulus-response model only |
| Banerjee, U.; Hine, J. “Identifying the underlying constructs linking urban form and travel behaviour using a grounded theory approach”. Int. J. Environ. Sci. Technol. 11, 2217–2232 (2014). https://doi.org/10.1007/s13762-014-0585-0 | Does not meet inclusion criteria: focus groups only, no intervention |
| Barr, S.; Prillwitz, J. “Green travellers? Exploring the spatial context of sustainable mobility styles”. Applied Geography, Volume 32, Issue 2, 798-809 (2012). https://doi.org/10.1016/j.apgeog.2011.08.002 | Does not meet inclusion criteria: method included 1. focus group, 2. survey, 3. focus group to explore responses to three travel policy scenarios, intention-based willingness only, no PEB intervention |
| Barr, S.; Prillwitz, J. “A Smarter Choice? Exploring the Behaviour Change Agenda for Environmentally Sustainable Mobility”. Environment and Planning C: Government and Policy, Volume 32, Issue 1, 1-19 (2014). https://doi.org/10.1068/c1201 | Does not meet inclusion criteria: same research project and methodological approach as Barr (2012) |
| Barrett, B. et al. “Mindful Climate Action: Health and Environmental Co-Benefits from Mindfulness-Based Behavioral Training”. Sustainability 2016, 8, 1040 (2016). https://doi.org/10.3390/su8101040 | Does not meet inclusion criteria: study protocol of planned intervention, no intervention conducted and no outcomes measured at time of publication |
| Bel, G.; Rosell, J. “The impact of socioeconomic characteristics on CO2 emissions associated with urban mobility: Inequality across individuals”. Energy Economics, Volume 64, 251-261 (2017). https://doi.org/10.1016/j.eneco.2017.04.002 | Does not meet inclusion criteria: econometric analysis, i.e. emission estimation based on survey, no PEB intervention |
| Bergquist M.; Nilsson A.; Hansla A. “Contests versus Norms: Implications of Contest-Based and Norm-Based Intervention Techniques”. Frontiers in Psychology, Volume 8, 2046 (2017). https://doi.org/10.3389/fpsyg.2017.02046 | Does not meet inclusion criteria: framing experiment to measure intentions only, no PEB intervention |
| Bodnar, P. et al. “Underwriting 1.5°C: competitive approaches to financing accelerated climate change mitigation”. Climate Policy, 18:3, 368-382 (2018). https://doi.org/10.1080/14693062.2017.1389687 | Does not meet inclusion criteria: focus on financial sector; no PEB intervention in individuals |
| Bolderdijk, J.W. et al. „Values Determine the (In)Effectiveness of Informational Interventions in Promoting Pro-Environmental Behavior”. PLoS ONE 8(12): e83911 (2013). https://doi.org/10.1371/journal.pone.0083911 | Does not meet inclusion criteria: framing experiment to measure values and intentions only, no PEB intervention |
| Brazil, W.; Kallbekken, S.; Sælen, H.; Carroll, J. „The role of fuel cost information in new car sales”. Transportation Research Part D: Transport and Environment, Volume 74, 93-103 (2019). https://doi.org/10.1016/j.trd.2019.07.022 | Does not meet inclusion criteria: discrete choice experiment (reframing of fuel cost information) only, no PEB intervention |
| Cai, S. et al. “Determinants of intention and behavior of low carbon commuting through bicycle-sharing in China”. Journal of Cleaner Production, Volume 212, 602-609 (2019). https://doi.org/10.1016/j.jclepro.2018.12.072 | Does not meet inclusion criteria: questionnaire to measure intentions only, no PEB intervention |
| Castán Broto, V.; Bulkeley, H. “A survey of urban climate change experiments in 100 cities”. Global Environmental Change, Volume 23, Issue 1, 92-102 (2013). https://doi.org/10.1016/j.gloenvcha.2012.07.005 | Does not meet inclusion criteria: development of database about climate change experiments, no PEB intervention |
| Chance, P.; Heward, W. L. “Climate change: meeting the challenge”. The Behavior analyst, 33(2), 197–206 (2010). https://doi.org/10.1007/BF03392219 | Does not meet inclusion criteria: mentioning of projects as examples without detailed descriptions regarding, e.g. study population, duration and outcomes |
| Chatterton, P. “The UK's first ecological, affordable cohousing community”. Int J Urban Reg Res, 37: 1654-1674 (2013).  https://doi.org/10.1111/1468-2427.12009 | Does not meet inclusion criteria: discussion of lessons learned from project’s development phase; no outcomes described |
| Chen, X. et al. “Sympathy for the environment predicts green consumerism but not more important environmental behaviours related to domestic energy use”. Environmental Conservation, 43(2), 140-147 (2016). https://doi.org/10.1017/S0376892915000351 | Does not meet inclusion criteria: mail-in surveys only, no PEB intervention |
| Chen, Y.; Sun, Y.; Wang, C. “Influencing Factors of Companies’ Behavior for Mitigation: A Discussion within the Context of Emission Trading Scheme”. Sustainability 2018, 10, 414 (2018). https://doi.org/10.3390/su10020414 | Does not meet inclusion criteria: online questionnaires only, no PEB intervention |
| Croci, E. et al. “Urban CO2 mitigation strategies under the Covenant of Mayors: An assessment of 124 European cities”. Journal of Cleaner Production, Volume 169, 161-177 (2017).  https://doi.org/10.1016/j.jclepro.2017.05.165 | Does not meet inclusion criteria: analysis and categorisation of policies for intended emission reductions without description of outcomes and impact |
| Darby, H. et al. “Influence of occupants’ behaviour on energy and carbon emission reduction in a higher education building in the UK”. Intelligent Buildings International, 8:3, 157-175 (2016). https://doi.org/10.1080/17508975.2016.1139535 | Full-text not available to review’s authors |
| Davies, Z. G.; Armsworth, P.R. “Making an impact: The influence of policies to reduce emissions from aviation on the business travel patterns of individual corporations”. Energy Policy, Volume 38, Issue 12, 7634-7638 (2010). https://doi.org/10.1016/j.enpol.2010.09.007 | Does not meet inclusion criteria: estimations of policy scenarios’ impact on CO2 emissions based on web-based questionnaire, no PEB intervention |
| de Boer, J.; de Witt, A.; Aiking, H. “Help the climate, change your diet: A cross-sectional study on how to involve consumers in a transition to a low-carbon society”. Appetite, Volume 98, 19-27 (2016). https://doi.org/10.1016/j.appet.2015.12.001 | Does not meet inclusion criteria: questionnaire to measure intention-based willingness only, no PEB intervention |
| Delina, L.L.; Diesendorf, M. „Is wartime mobilisation a suitable policy model for rapid national climate mitigation?”. Energy Policy, Volume 58, 371-380 (2013). https://doi.org/10.1016/j.enpol.2013.03.036 | Does not meet inclusion criteria: historical analysis how war times can motivate change, no PEB intervention |
| Delina, L.L.; Diesendorf, M.; Merson, J. “Strengthening the climate action movement:  strategies from histories”. Carbon Management, 5:4, 397-409 (2014). https://doi.org/10.1080/17583004.2015.1005396 | Does not meet inclusion criteria: historical analysis of mechanisms that achieved effective social change in the past, no PEB intervention |
| Dietz, T.; Stern, P.C.; Weber, E.U. “Reducing Carbon-Based Energy Consumption through Changes in Household Behavior”. Daedalus Volume 142:1, 78-89 (2013). https://doi.org/10.1162/DAED_a_00186 | Does not meet inclusion criteria: studies mentioned as examples without detailed descriptions regarding, e.g. study population, duration and outcomes |
| Dominelli, L. “Climate change: social workers' roles and contributions to policy debates and interventions”. International Journal of Social Welfare, 20: 430-438 (2011). https://doi.org/10.1111/j.1468-2397.2011.00795.x | Does not meet inclusion criteria: two case studies mentioned, but without description of duration, impact on emissions or behavior |
| Dowd, A-M.; Hobman, E. “Mobilizing citizens for a low and clean energy future”. Current Opinion in Environmental Sustainability, Volume 5, Issue 2, 191-196 (2013).  https://doi.org/10.1016/j.cosust.2013.04.005 | Does not meet inclusion criteria: studies mentioned as examples without descriptions regarding methodology, no PEB intervention |
| Drews, S.; van den Bergh, J.C.J.M. „What explains public support for climate policies? A review of empirical and experimental studies”. Climate Policy, 16:7, 855-876 (2016).  https://doi.org/10.1080/14693062.2015.1058240 | Does not meet inclusion criteria: studies using preferences, choice experiments or opinion polls mentioned as examples, no PEB interventions |
| Dubois, G. et al. “It starts at home? Climate policies targeting household consumption and behavioral decisions are key to low-carbon futures”. Energy Research & Social Science, Volume 52, 144-158 (2019). https://doi.org/10.1016/j.erss.2019.02.001 | Does not meet inclusion criteria: household analysis using questionnaires, simulations and qualitative interviews, no PEB intervention |
| Eagle, L. et al. “Social marketing strategies for renewable energy transitions”. Australasian Marketing Journal (AMJ), Volume 25, Issue 2, 141-148 (2017). https://doi.org/10.1016/j.ausmj.2017.04.006 | Does not meet inclusion criteria: questionnaire on electricity usage and attitudes towards renewable energy only, no PEB intervention |
| Eijgelaar, E. “Voluntary Carbon Offsets a Solution for Reducing Tourism Emissions? Assessment of Communication Aspects and Mitigation Potential”. European Journal of Transport and Infrastructure Research, [S.l.], v. 11, n. 3 (2011). https://doi.org/10.18757/ejtir.2011.11.3.2933. | Does not meet inclusion criteria: website analysis in tourism sector and assessments of awareness of air travel impacts and willingness to pay, no PEB intervention |
| Elrick-Barr, C.E. et al. “How are coastal households responding to climate change?”. Environmental Science & Policy, Volume 63, 177-186 (2016). https://doi.org/10.1016/j.envsci.2016.05.013 | Does not meet inclusion criteria: questionnaire and semi-structured interviews to assess household responses to climate change, no PEB Intervention |
| Evans, L.; Milfont, T.L.; Lawrence, J. “Considering local adaptation increases willingness to mitigate”. Global Environmental Change, Volume 25, 69-75 (2014). https://doi.org/10.1016/j.gloenvcha.2013.12.013 | Does not meet inclusion criteria: survey to measure willingness to mitigate; no PEB intervention |
| Fankhauser, S. “A Practitioner’s Guide to a Low-Carbon Economy: Lessons from the UK”. Climate Policy, 13(3): 345-362 (2013) https://doi.org/10.1080/14693062.2013.749124 | Does not meet inclusion criteria: list of existing policies, no description of PEB intervention and respective outcomes |
| Fenton, P. “Sustainable mobility in the low carbon city: Digging up the highway in Odense, Denmark”. Sustainable Cities and Society, Volume 29, 203-210 (2017). https://doi.org/10.1016/j.scs.2016.11.006 | Does not meet inclusion criteria: focusses on interviews and planned restructuring of urban mobility, no detailed description of PEB intervention or outcomes |
| Field, A. et al. “Encountering bikelash: Experiences and lessons from New Zealand communities”. Journal of Transport & Health, Volume 11, 130-140 (2018). https://doi.org/10.1016/j.jth.2018.10.003 | Does not meet inclusion criteria: bike lanes are an intervention, but paper focusses on interviews about emotions and support regarding implementation, no detailed description of PEB impact and outcomes |
| Fink, H.S. “Promoting behavioral change towards lower energy consumption in the building sector”. Innovation: The European Journal of Social Science Research, 24:1-2, 7-26 (2011).  https://doi.org/10.1080/13511610.2011.586494 | Does not meet inclusion criteria: theoretical approach with studies mentioned as examples, no detailed description of PEB intervention or outcomes |
| Fischer, A. et al. „Energy use, climate change and folk psychology: Does sustainability have a chance? Results from a qualitative study in five European countries”. Global Environmental Change, Volume 21, Issue 3, 1025-1034 (2011). https://doi.org/10.1016/j.gloenvcha.2011.04.008 | Does not meet inclusion criteria: qualitative, semi-structured interviews and modelling approaches only; no PEB intervention |
| Fleiß, E. et al. „Money, not morale: The impact of desires and beliefs on private investment in photovoltaic citizen participation initiatives”. Journal of Cleaner Production, Volume 141, 920-927 (2017). https://doi.org/10.1016/j.jclepro.2016.09.123 | Does not meet inclusion criteria: questionnaire and expert interviews only; no PEB intervention |
| Frederiks, E.R.; Stenner, K.; Hobman, E.V. „Household energy use: Applying behavioural economics to understand consumer decision-making and behaviour". Renewable and Sustainable Energy Reviews, Volume 41, 1385-1394 (2015). https://doi.org/10.1016/j.rser.2014.09.026 | Does not meet inclusion criteria: theoretical approach, no PEB intervention |
| Fudge, S.; Peters, M. “Behaviour Change in the UK Climate Debate: An Assessment of Responsibility, Agency and Political Dimensions”. Sustainability 2011, 3, 789-808 (2011). https://doi.org/10.3390/su3060789 | Does not meet inclusion criteria: theoretical approach using focus groups, no PEB intervention |
| Gampfer, R. “Do individuals care about fairness in burden sharing for climate change mitigation? Evidence from a lab experiment”. Climatic Change 124, 65–77 (2014).  https://doi.org/10.1007/s10584-014-1091-6 | Does not meet inclusion criteria: lab experiment to answer research question about fairness, no PEB intervention aiming at behavior change |
| Gans et al. “Multilevel approaches to increase fruit and vegetable intake in low-income housing communities: final results of the ‘Live Well, Viva Bien’ cluster-randomized trial”. International Journal of Behavioral Nutrition and Physical Activity, 15:80 (2018). https://doi.org/10.1186/s12966-018-0704-2 | Does not meet inclusion criteria: intervention to increase fruit and vegetable intake with focus on health not sustainability, no PEB-related outcomes measured |
| Glover, A.; Strengers, Y.; Lewis, T. "Sustainability and academic air travel in Australian universities". International Journal of Sustainability in Higher Education, Vol. 19 No. 4, 756-772 (2018). https://doi.org/10.1108/IJSHE-08-2017-0129 | Full-text not available to review’s authors |
| Grabow, M. et al. “Mindfulness and Climate Change Action: A Feasibility Study”. Sustainability 2018, 10, 1508 (2018).  https://doi.org/10.3390/su10051508 | Does not meet inclusion criteria: as-is analysis without possibility of comparing before- and after-PEB, no behavior change or its impact and sustainability measurable |
| Grasso, A.C. et al. “Effect of food-related behavioral activation therapy on food intake and the environmental impact of the diet: results from the MooDFOOD prevention trial”. Eur J Nutr 59, 2579–2591 (2020). https://doi.org/10.1007/s00394-019-02106-1 | Does not meet inclusion criteria: RCT to investigate two different nutritional strategies for the prevention of depression; environmental impact of diet was calculated but not considered in intervention design and no information regarding this was given to participants, consequently, it is not a PEB intervention |
| Green, F.; Gambhir, A. “Transitional assistance policies for just, equitable and smooth low-carbon transitions: who, what and how?”. Climate Policy, 20:8, 902-921 (2020). https://doi.org/10.1080/14693062.2019.1657379 | Does not meet inclusion criteria: typology of policy-instruments, no focus on PEB or PEB intervention |
| Gromet, D.M.; Kunreuther, H.; Larrick, R.P. “Political ideology affects energy-efficiency attitudes and choices” PNAS June 4, 2013 110 (23) 9314-9319 (2013). https://doi.org/10.1073/pnas.1218453110 | Does not meet inclusion criteria: two choice experiments on preferences, no PEB intervention for sustainable behavior change |
| Hafner, R.J.; Elmes, D.; Read, D. “Promoting behavioural change to reduce thermal energy demand in households: A review”. Renewable and Sustainable Energy Reviews, Volume 102, 205-214 (2019). https://doi.org/10.1016/j.rser.2018.12.004 | Does not meet inclusion criteria: review to identify factors influencing PEB, no methodical description how literature was identified, only categorization of interventions without descriptions of interventions and their outcomes |
| Haider, S.W., Zhuang, G. & Ali, S. Identifying and bridging the attitude-behavior gap in sustainable transportation adoption. J Ambient Intell Human Comput 10, 3723–3738 (2019).  https://doi.org/10.1007/s12652-019-01405-z | Does not meet inclusion criteria: expert interviews to list barriers of electric vehicle adoption in India; no PEB intervention |
| Hall, C.M. “Intervening in academic interventions: framing social marketing's potential for successful sustainable tourism behavioural change”. Journal of Sustainable Tourism, 24:3, 350-375 (2016) https://doi.org/10.1080/09669582.2015.1088861 | Does not meet inclusion criteria: theoretical approach to sustainable tourism, no PEB intervention |
| Hankey, S. et al. “Using objective measures of stove use and indoor air quality to evaluate a cookstove intervention in rural Uganda”. Energy for Sustainable Development, Volume 25, 67-74 (2015). https://doi.org/10.1016/j.esd.2014.12.007 | Does not meet inclusion criteria: PEB-related intervention focusing on air pollution, but not on acceptance and how/ if intervention lead to behavior change |
| Hayashi, K.; Hondo, H.; Moriizumi, Y. “Preference Construction Processes for Renewable Energies: Assessing the Influence of Sustainability Information and Decision Support Methods”. Sustainability 2016, 8(11), 1114 (2016). https://doi.org/10.3390/su8111114 | Does not meet inclusion criteria: framing experiment measuring preferences only, no PEB intervention |
| Helm, D. “Government failure, rent-seeking, and capture: the design of climate change policy”. Oxford Review of Economic Policy, Volume 26, Issue 2, Summer 2010, 182–196 (2010). https://doi.org/10.1093/oxrep/grq006 | Does not meet inclusion criteria: historical economic policy analysis, no PEB intervention or focus on behavior change |
| Herrmann, A.; Sauerborn, R.; Nilsson, M. “The Role of Health in Households’ Balancing Act for Lifestyles Compatible with the Paris Agreement—Qualitative Results from Mannheim, Germany”. Int. J. Environ. Res. Public Health 2020, 17, 1297 (2020). https://doi.org/10.3390/ijerph17041297 | Does not meet inclusion criteria: choice-experiment to measure preferences with questions relating to the reasons for choices, no PEB intervention |
| Higham, J. et al. “Climate change, tourist air travel and radical emissions reduction”. Journal of Cleaner Production, Volume 111, Part B, 336-347 (2016). https://doi.org/10.1016/j.jclepro.2014.10.100 | Does not meet inclusion criteria: as-is analysis based on interviews only, no PEB intervention |
| Hobman, E.V.; Ashworth, P. “Public support for energy sources and related technologies: The impact of simple information provision”. Energy Policy, Volume 63, 862-869 (2013). https://doi.org/10.1016/j.enpol.2013.09.011 | Does not meet inclusion criteria: online survey to measure preferences only, no PEB intervention |
| Hobson, K.; Hamilton, J.; Mayne, R. “Monitoring and evaluation in UK low-carbon community groups: benefits, barriers and the politics of the local”. Local Environment, 21:1, 124-136 (2016). https://doi.org/10.1080/13549839.2014.928814 | Does not meet inclusion criteria: theoretical approach with studies mentioned as examples, no detailed description of PEB interventions or outcomes |
| Holloway, T.; Salter, A. M.; McCullough, F. S. “Dietary intervention to reduce meat intake by 50% in University students – a pilot study”. Proceedings of the Nutrition Society (2012), 71 (OCE2), E164 (2012). https://doi.org/10.1017/S0029665112002212 | Does not meet inclusion criteria: weaknesses in study’s methods, e.g. study period too short for measured outcomes weight and BMI, no focus on PEB outcomes |
| Hopkins, D. et al. “Academic mobility in the Anthropocene era: a comparative study of university policy at three New Zealand institutions”. Journal of Sustainable Tourism, 24:3, 376-397 (2016). https://doi.org/10.1080/09669582.2015.1071383 | Does not meet inclusion criteria: content analysis of universities’ policies only, no measurement of implementation/ acceptance or possible behavior change |
| Howden-Chapman, P.L. et al. “Urban interventions: understanding health co-benefits”. Proceedings of the Institution of Civil Engineers Urban Design and Planning 168 August 2015 Issue DP4, 196–203 (2015). https://doi.org/10.1680/udap.14.00049 | Does not meet inclusion criteria: focus on health benefits after application of technology or changes in infrastructure, no PEB intervention |
| Howden-Chapman, P.L. et al. “Evaluating natural experiments to measure the co-benefits of urban policy interventions to reduce carbon emissions in New Zealand”. Science of The Total Environment, Volume 700, 134408 (2020). https://doi.org/10.1016/j.scitotenv.2019.134408 | Does not meet inclusion criteria: description of study evaluation design only; none of the natural experiments explicitly focused on reducing carbon emissions, no PEB intervention |
| Howell, R.A. “Carbon management at the household level: a definition of carbon literacy and three mechanisms that increase it”. Carbon Management, 9:1, 25-35 (2018). https://doi.org/10.1080/17583004.2017.1409045 | Implicit duplicate since it is a summary of Howell (2011) and Howell (2012), which were already included in review |
| Isley, S.C. et al. “Online purchasing creates opportunities to lower the life cycle carbon footprints of consumer products”. PNAS 113 (35) 9780-9785 (2016). https://doi.org/10.1073/pnas.1522211113 | Does not meet inclusion criteria: choice experiment only, no PEB intervention |
| Ivanova, D. et al. “Carbon mitigation in domains of high consumer lock-in”. Global Environmental Change, Volume 52, 117-130 (2018). https://doi.org/10.1016/j.gloenvcha.2018.06.006 | Does not meet inclusion criteria: survey on factors and calculations based on those surveys, no PEB intervention |
| Jadro, B.V. “Research Note - The Use Of An Onboard Diagnostic Device To Provide Feedback On Driving Behaviors Related To Fuel Economy” Behavior and Social Issues, 26, 190-193 (2017). https://doi.org/10.5210/bsi.v.26i0.6891 | Does not meet inclusion criteria: comparison of technical meter devices to give feedback in driving interventions; no PEB intervention |
| Jariyasunant, J. et al. “Quantified Traveler: Travel Feedback Meets the Cloud to Change Behavior”. Journal of Intelligent Transportation Systems, 19:2, 109-124 (2015). https://doi.org/10.1080/15472450.2013.856714 | Full-text not available to review’s authors |
| Jensen, C.L. et al. “Towards a practice-theoretical classification of sustainable energy consumption initiatives: Insights from social scientific energy research in 30 European countries”. Energy Research & Social Science, Volume 45, 297-306 (2018). https://doi.org/10.1016/j.erss.2018.06.025 | Does not meet inclusion criteria: practice-theoretical classification of sustainable energy consumption initiatives; no detailed description of PEB intervention |
| Jiang, P. et al. “Building low carbon communities in China: The role of individual’s behaviour change and engagement”. Energy Policy, Volume 60, 611-620 (2013). https://doi.org/10.1016/j.enpol.2013.05.017 | Does not meet inclusion criteria: as-is analysis of some general principles to change peoples’ behavior; no PEB intervention |
| Jiang, P. et al. “Individual environmental behavior: A key role in building low-carbon communities in China“. Front. Energy 2018, 12(3): 456–465 (2018). https://doi.org/10.1007/s11708-018-0566-y | Does not meet inclusion criteria: questionnaires for as-is analysis on behaviors and habits; methodical unclear how to trace changes back to a specific policy |
| Junghans, L.; Kreft, S.; Welp, M. “Inclusive Visions for Urban Transitions: Lessons from stakeholder dialogues in Asian medium sized cities”. Sustainable Cities and Society, Volume 42, 512-520 (2018). https://doi.org/10.1016/j.scs.2018.08.003 | Does not meet inclusion criteria: stakeholder dialogues without measurement of outcomes, no follow-up, no direct PEB intervention |
| Kaklamanou, D. et al. “Using Public Transport Can Make Up for Flying Abroad on Holiday: Compensatory Green Beliefs and Environmentally Significant Behavior”. Environment and Behavior 2015, Vol. 47(2) 184–204 (2015). https://doi.org/10.1177/0013916513488784 | Does not meet inclusion criteria: online questionnaire for as-is-analysis to assess participants’ behavior, worldviews, identity and beliefs; no PEB intervention |
| Kammerlander, M. et al. “How does a social practice perspective add to the development of policy instruments to reduce consumption-based CO2 emissions? A case study of Austria”. Climate Policy, 20:3, 323-340 (2020). https://doi.org/10.1080/14693062.2020.1727830 | Full-text not available to review’s authors |
| Kantenbacher, J. et al. “Public attitudes about climate policy options for aviation”. Environmental Science & Policy, Volume 81, 46-53 (2018). https://doi.org/10.1016/j.envsci.2017.12.012 | Does not meet inclusion criteria: online survey to measure environmental attitudes for as-is-analysis; no PEB intervention |
| Katzeff, C. et al. “Exploring Sustainable Practices in Workplace Settings through Visualizing Electricity Consumption”. ACM Trans. Comput.-Hum. Interact. 20, 5, Article 31 (2013). https://doi.org/10.1145/2501526 | Does not meet inclusion criteria: feedback study focusing on technical solution for feedback provision not behavior change |
| Klenert, D. et al. „Making carbon pricing work for citizens”. Nature Clim Change 8, 669–677 (2018). https://doi.org/10.1038/s41558-018-0201-2 | Does not meet inclusion criteria: analysis of carbon tax reform and how successful it was, focusing on country-level; no outcomes measured on individual behavior or PEB |
| Klepacka, A.M.; Florkowski, W.J.; Meng, T. “Clean, accessible, and cost-saving: Reasons for rural household investment in solar panels in Poland”. Resources, Conservation and Recycling, Volume 139, 338-350 (2018). https://doi.org/10.1016/j.resconrec.2018.09.004 | Does not meet inclusion criteria: questionnaire to assess views about solar panel adoptions; impact of subsidies on solar energy and PEB were not measured; no PEB intervention |
| Laicane, I. et al. “Development of methodology for the assessment of changes in household electricity consumption and calculation of CO2 emissions”. International Journal of Global Warming (IJGW), Vol. 8, No. 1 (2015). https://doi.org/10.1504/IJGW.2015.071582 | Full-text not available to review’s authors |
| Laitala, K.; Klepp, I.G.; Boks, C. “Changing laundry habits in Norway”. International Journal of Consumer Studies, 36: 228-237 (2012).  https://doi.org/10.1111/j.1470-6431.2011.01081.x | Does not meet inclusion criteria: surveys and in-depth interviews regarding laundry habits only; no PEB intervention |
| Leach, J.M. et al. “The Liveable Cities Method: Establishing the 2 Case for Transformative Change”. Proceedings of the Institution of Civil Engineers - Engineering Sustainability 2020 173:1, 8-19 (2020). https://doi.org/10.1680/jensu.18.00028 | Does not meet inclusion criteria: description of method to develop interventions and strategies for a sustainable future city; no PEB intervention |
| Lee, P.-S. et al. “Using Episodic Future Thinking to Pre-Experience Climate Change Increases Pro-Environmental Behavior”. Environment and Behavior, Vol. 52(1) 60–81 (2020).  https://doi.org/10.1177/0013916518790590 | Does not meet inclusion criteria: choice experiments to influence risk perception regarding climate change; no PEB intervention |
| Lin, S.-P. “Raising Public Awareness: The Role of the Household Sector in Mitigating Climate Change”. Int. J. Environ. Res. Public Health 2015, 12, 13162-13178 (2015). https://doi.org/10.3390/ijerph121013162 | Does not meet inclusion criteria: questionnaires and interviews to examine intentions regarding PEB only; no PEB intervention |
| Macmillan, A.K. et al. “Controlled before-after intervention study of suburb-wide street changes to increase walking and cycling: Te Ara Mua-Future Streets study design”. BMC Public Health 18, 850 (2018). https://doi.org/10.1186/s12889-018-5758-1 | Does not meet inclusion criteria: study design/ protocol only, no outcomes measured |
| Marteau T.M. “Towards environmentally sustainable human behaviour: targeting non-conscious and conscious processes for effective and acceptable policies”. Phil. Trans. R. Soc. A.37520160371 (2017). http://doi.org/10.1098/rsta.2016.0371 | Does not meet inclusion criteria: theoretical description of PEB intervention research; no own PEB intervention |
| Morten, A.; Gatersleben, B.; Jessop, D.C. “Staying grounded? Applying the theory of planned behaviour to explore motivations to reduce air travel”. Transportation Research Part F: Traffic Psychology and Behaviour, Volume 55, 297-305 (2018). https://doi.org/10.1016/j.trf.2018.02.038 | Does not meet inclusion criteria: online questionnaire for as-is-analysis regarding intentions only; no PEB intervention |
| Moser, C.; Blumer, Y.; Hille, S.L. “E-bike trials' potential to promote sustained changes in car owners mobility habits”. Environ. Res. Lett. 13 044025 (2018). https://doi.org/10.1088/1748-9326/aaad73 | Does not meet inclusion criteria: study did not measure actual habits or behavior but rather habitual associations |
| Otto, I.M. et al. “Social tipping dynamics for stabilizing Earth’s climate by 2050”. Proceedings of the National Academy of Sciences Feb 2020, 117 (5) 2354-2365 (2020). https://doi.org/10.1073/pnas.1900577117 | Does not meet inclusion criteria: proposal of policy-based interventions based on expert workshop; no detailed description of PEB intervention or outcomes |
| Paddock, J. “Household consumption and environmental change: Rethinking the policy problem through narratives of food practice”. Journal of Consumer Culture Vol. 17(1) 122–139 (2017). https://doi.org/10.1177/1469540515586869 | Does not meet inclusion criteria: questionnaire/ interview; no PEB intervention |
| Palm, J.; Ellegård, K. “Visualizing energy consumption activities as a tool for developing effective policy”. International Journal of Consumer Studies, 35: 171-179 (2011). https://doi.org/10.1111/j.1470-6431.2010.00974.x | Does not meet inclusion criteria: software for time diaries to observe behavior; no PEB intervention |
| Parajuli, R. “Looking into the Danish energy system: Lesson to be learned by other communities”. Renewable and Sustainable Energy Reviews, Volume 16, Issue 4, 2191-2199 (2012). https://doi.org/10.1016/j.rser.2012.01.045 | Does not meet inclusion criteria: assessment of policies in Denmark regarding energy; no PEB intervention |
| Parant, A. et al. “Raising Students Awareness to Climate Change: An Illustration With Binding Communication”. Environment and Behavior, Vol. 49(3) 339–353 (2017). https://doi.org/10.1177/0013916516629191 | Does not meet inclusion criteria: information campaign to raise awareness, but only willingness measured, not behavior change |
| Patterson, J.L. “Evaluation of a Regional Retrofit Programme to Upgrade Existing Housing Stock to Reduce Carbon Emissions, Fuel Poverty and Support the Local Supply Chain”. Sustainability 2016, 8(12), 1261 (2016). https://doi.org/10.3390/su8121261 | Does not meet inclusion criteria: focus on technical aspects of retrofit; no PEB intervention |
| Pearson, D.; Friel, S.; Lawrence, M. “Building environmentally sustainable food systems on informed citizen choices: evidence from Australia”. Biological Agriculture & Horticulture, 30:3, 183-197 (2014). https://doi.org/10.1080/01448765.2014.890542 | Full-text not available to review’s authors |
| Pedros-Perez, G.; Martínez-Jiménez, P.; Aparicio-Martinez, P. “The potential of car advertising in pursuing transport policy goals: Code of good practices in the Spanish context”. Transportation Research Part D: Transport and Environment, Volume 72, 312-332 (2019). https://doi.org/10.1016/j.trd.2019.05.010 | Does not meet inclusion criteria: assessment how car advertisments changed over time and due to climate change awareness in society; no PEB intervention |
| Penz, E.; Hartl, B.; Hofmann, E. “Explaining consumer choice of low carbon footprint goods using the behavioral spillover effect in German-speaking countries”. Journal of Cleaner Production, Volume 214, 429-439 (2019). https://doi.org/10.1016/j.jclepro.2018.12.270 | Does not meet inclusion criteria: self-reported data on intention to perform easy versus difficulty sustainable behavior only; no focus on PEB intervention |
| Perera, P. et al. “Scenario-based economic and environmental analysis of clean energy incentives for households in Canada: Multi criteria decision making approach”. Journal of Cleaner Production, Volume 198, 170-186 (2018). https://doi.org/10.1016/j.jclepro.2018.07.014 | Does not meet inclusion criteria: computed scenario-based assessments only; no PEB intervention |
| Pollard, C. E. “Up close and personal The value of feedback in implementing an individual energy-saving adaptation”, International Journal of Sustainability in Higher Education, Vol. 17, No. 1, 68-85 (2016). https://doi.org/10.1108/IJSHE-03-2014-0043 | Full-text not available to review’s authors |
| Rehman, I.H. et al. „Distribution of improved cook stoves: analysis of field experiments using strategic niche management theory”. Sustain Sci 7, 227–235 (2012). https://doi.org/10.1007/s11625-012-0162-8 | Does not meet inclusion criteria: test and optimization of cookstove technology and general questionnaire on cook stoves; no PEB intervention on adaption or behavior change |
| Robèrt, M. “Strategic travel planning toward future emission targets—A comparative analysis of 20 Swedish municipalities applying the CERO model”. International Journal of Sustainable Transportation, 11:5, 330-341 (2017). https://doi.org/10.1080/15568318.2016.1232452 | Full-text not available to review’s authors |
| Rooney-Varga, J.N. et al. “The Climate Action Simulation”. Simulation & Gaming 2020, Vol. 51(2) 114–140 (2020). https://doi.org/10.1177/1046878119890643 | Does not meet inclusion criteria: educative simulation and survey to measure willingness to act politically and change behavior but no follow-up and no assessment if game lead to actual behavior change |
| Salo, M. et al. “Tailored advice and services to enhance sustainable household consumption in Finland”. Journal of Cleaner Production, Volume 121, 200-207 (2016). https://doi.org/10.1016/j.jclepro.2016.01.092. | Does not meet inclusion criteria: four action models without detailed description of methods; or PEB outcomes |
| Salo, M.; Mattinen-Yuryev, M.K.; Nissinen, A. “Opportunities and limitations of carbon footprint calculators to steer sustainable household consumption – Analysis of Nordic calculator features”. Journal of Cleaner Production, Volume 207, 658-666 (2019). https://doi.org/10.1016/j.jclepro.2018.10.035 | Does not meet inclusion criteria: examination of calculation tools and interviews with calculator hosts to study calculator features and hosts' experiences; no PEB intervention |
| Schröder, P. et al. “Advancing sustainable consumption and production in cities - A transdisciplinary research and stakeholder engagement framework to address consumption-based emissions and impacts”. Journal of Cleaner Production, Volume 213, 114-125 (2019). https://doi.org/10.1016/j.jclepro.2018.12.050 | Does not meet inclusion criteria: examples of initiatives but no detailed description of methods (e.g. number of participants) or outcomes |
| Schultz, N.R.; Kohn, C.S.; Musto, A. “Examination Of A Multi-Element Intervention On College Students’ Electricity Consumption In On-Campus Housing“. Behav. Intervent. 32: 79–90 (2017).  https://doi.org/10.1002/bin.1463 | Does not meet inclusion criteria: intervention but no measurement of actual behavior, prohibiting deduction of behavior changes |
| Scott, M.G.; Lawson, R. “The road code: encouraging more efficient driving practices in New Zealand”. Energy Efficiency 11, 1617–1626 (2018). https://doi.org/10.1007/s12053-017-9538-z | Does not meet inclusion criteria: focus group approach, no direct PEB intervention |
| Seebauer, S.; Fleiß, J.; Schweighart, M. “A Household Is Not a Person: Consistency of Pro-Environmental Behavior in Adult Couples and the Accuracy of Proxy-Reports“. Environment and Behavior, Vol. 49(6) 603–637 (2017). https://doi.org/10.1177/0013916516663796 | Does not meet inclusion criteria: interviews of households for as-is-analysis; no PEB intervention |
| Seidl, R.; Moser, C.; Blumer Y. “Navigating behavioral energy sufficiency. Results from a survey in Swiss cities on potential behavior change”. PLoS ONE 12(10): e0185963 (2017). https://doi.org/10.1371/journal.pone.0185963 | Does not meet inclusion criteria: online survey for as-is-analysis; no PEB intervention |
| Semenza, J.C.; Ploubidis, G.B.; George, L.A. “Climate change and climate variability: personal motivation for adaptation and mitigation”. Environ Health 10, 46 (2011). https://doi.org/10.1186/1476-069X-10-46 | Does not meet inclusion criteria: telephone surveys on attitudes about climate change for as-is-analysis; no PEB intervention |
| Shalev, I. “The climate change problem: promoting motivation for change when the map is not the territory”. Frontiers in Psychology, Volume 6, 131 (2015). https://doi.org/10.3389/fpsyg.2015.00131 | Does not meet inclusion criteria: theoretical approach on making decisions about climate change problem; no PEB intervention |
| Sharp, L.; Macrorie, R.; Turner, A. “Resource efficiency and the imagined public: Insights from cultural theory”. Global Environmental Change, Volume 34, 196-206 (2015). https://doi.org/10.1016/j.gloenvcha.2015.07.001 | Does not meet inclusion criteria: conceptual framework for assessing resource governance; no detailed description of PEB intervention |
| Sifakis, N. et al. “Analysis of the Energy Consumption Behavior of European RES Cooperative Members”. Energies, 12(6), 970 (2019). https://doi.org/10.3390/en12060970 | Does not meet inclusion criteria: no detailed description of PEB intervention |
| Skouteris, H.et al. “Promoting obesity prevention together with environmental sustainability”. Health Promotion International, Volume 29(3), 454-462 (2014). https://doi.org/10.1093/heapro/dat007 | Does not meet inclusion criteria: no detailed description of methods nor PEB interventions |
| Sparkman, G.; Attari, S.Z. “Credibility, communication, and climate change: How lifestyle inconsistency and do-gooder derogation impact decarbonization advocacy”. Energy Research & Social Science, Volume 59, 101290 (2020). https://doi.org/10.1016/j.erss.2019.101290 | Does not meet inclusion criteria: questionnaire after framing experiment only, no PEB intervention |
| Steinhorst, J.; Klöckner, C.A.; Matthies, E. “Saving electricity – For the money or the environment? Risks of limiting pro-environmental spillover when using monetary framing”. Journal of Environmental Psychology, Volume 43, 125-135 (2015). https://doi.org/10.1016/j.jenvp.2015.05.012 | Does not meet inclusion criteria: online-questionnaires with tailored electricity saving tips; no focus on behavior change and no detailed description of PEB outcomes |
| Streimikiene, D.; Vveinhardt, J. “Community based social marketing for implementation of energy saving targets at local level”. Amfiteatru Economic, 17(39), 723-734 (2015). | Does not meet inclusion criteria: focus group intervention but weaknesses in methods, e.g. no description of the information given to the group and not all measured outcome variables are described |
| Swim, J.K.; Becker, J.C. “Country Contexts and Individuals’ Climate Change Mitigating Behaviors: A Comparison of U.S. Versus German Individuals’ Efforts to Reduce Energy Use”.Journal of Social Issues, Vol. 68, No. 3, 571—591 (2012). | Does not meet inclusion criteria: survey only, no PEB intervention |
| Thoyre, A. “Home climate change mitigation practices as gendered labor”. Women's Studies International Forum, Volume 78, 102314 (2020). https://doi.org/10.1016/j.wsif.2019.102314 | Does not meet inclusion criteria: theory analysis of semi-structured interviews; no PEB intervention |
| Tobler, C.; Visschers, V.H.M.; Siegrist, M. “Addressing climate change: Determinants of consumers' willingness to act and to support policy measures”. Journal of Environmental Psychology, Volume 32, Issue 3, 197-207 (2012). https://doi.org/10.1016/j.jenvp.2012.02.001 | Does not meet inclusion criteria: questionnaires for as-is-analysis; no PEB intervention |
| Unsworth, K.L.; McNeill, I. “Increasing Pro-Environmental Behaviors by Increasing Self-Concordance: Testing an Intervention”. Journal of Applied Psychology, 102 (1), 88-103 (2017). https://doi.org/10.1037/apl0000155 | Does not meet inclusion criteria: framing intervention to measure intentions only, but no PEB intervention |
| Webb, J. “Climate Change and Society: The Chimera of Behaviour Change Technologies”. Sociology, 46(1), 109–125 (2012).  https://doi.org/10.1177/0038038511419196 | Does not meet inclusion criteria: sociological critique of the consumer society model; no PEB intervention |
| Weiand, L. et al. “Climate change and air pollution: the connection between traffic intervention policies and public acceptance in a local context”. Environ. Res. Lett. 14 085008 (2019). https://doi.org/10.1088/1748-9326/ab3d81 | Does not meet inclusion criteria: weaknesses in description of methods |
| Winslott Hiselius L. “Can Mobility Management Campaigning Contribute to Pro-Environmental Behaviour in General? Development of an Analytical Tool”. PROMET 26(3):257-64 (2014). https://doi.org/10.7307/ptt.v26i3.1374 | Does not meet inclusion criteria: description of an analytical tool for analysing types of behavioural changes only, no PEB intervention |
| Wolske, K.S. et al. “Accelerating demand for residential solar photovoltaics: Can simple framing strategies increase consumer interest?”. Global Environmental Change, Volume 53, 68-77 (2018). https://doi.org/10.1016/j.gloenvcha.2018.08.005 | Does not meet inclusion criteria: framing intervention to measure preferences only, but no PEB intervention |
| York, J.G.; Vedula, S.; Lenox, M.J. “It’s Not Easy Building Green: The Impact of Public Policy, Private Actors, and Regional Logics on Voluntary Standards Adoption”. AMJ, 61, 1492–1523 (2018). https://doi.org/10.5465/amj.2015.0769 | Does not meet inclusion criteria: model and estimates, but no PEB intervention |
| Zen, I.S. et al. “Institutionalize waste minimization governance towards campus sustainability: A case study of Green Office initiatives in Universiti Teknologi Malaysia”. Journal of Cleaner Production, Volume 135, 1407-1422 (2016). https://doi.org/10.1016/j.jclepro.2016.07.053 | Does not meet inclusion criteria: weaknesses in description of methods |

Excluded: focus groups (interviews) only without intervention before/ after and measure of outcomes
